# Rapid Typing of *Coxiella burnetii*

**DOI:** 10.1371/journal.pone.0026201

**Published:** 2011-11-02

**Authors:** Heidie M. Hornstra, Rachael A. Priestley, Shalamar M. Georgia, Sergey Kachur, Dawn N. Birdsell, Remy Hilsabeck, Lauren T. Gates, James E. Samuel, Robert A. Heinzen, Gilbert J. Kersh, Paul Keim, Robert F. Massung, Talima Pearson

**Affiliations:** 1 Center for Microbial Genetics and Genomics, Northern Arizona University, Flagstaff, Arizona, United States of America; 2 Rickettsial Zoonoses Branch, Centers for Disease Control and Prevention, Atlanta, Georgia, United States of America; 3 Department of Microbial and Molecular Pathogenesis, Texas A&M Health Science Center, College Station, Texas, United States of America; 4 Laboratory of Intracellular Parasites, Rocky Mountain Laboratories, National Institute of Allergy and Infectious Diseases, National Institutes of Health, Hamilton, Montana, United States of America; 5 Pathogen Genomics Division, Translational Genomics Research Institute, Phoenix, Arizona, United States of America; Duke University Medical Center, United States of America

## Abstract

*Coxiella burnetii* has the potential to cause serious disease and is highly prevalent in the environment. Despite this, epidemiological data are sparse and isolate collections are typically small, rare, and difficult to share among laboratories as this pathogen is governed by select agent rules and fastidious to culture. With the advent of whole genome sequencing, some of this knowledge gap has been overcome by the development of genotyping schemes, however many of these methods are cumbersome and not readily transferable between institutions. As comparisons of the few existing collections can dramatically increase our knowledge of the evolution and phylogeography of the species, we aimed to facilitate such comparisons by extracting SNP signatures from past genotyping efforts and then incorporated these signatures into assays that quickly and easily define genotypes and phylogenetic groups. We found 91 polymorphisms (SNPs and indels) among multispacer sequence typing (MST) loci and designed 14 SNP-based assays that could be used to type samples based on previously established phylogenetic groups. These assays are rapid, inexpensive, real-time PCR assays whose results are unambiguous. Data from these assays allowed us to assign 43 previously untyped isolates to established genotypes and genomic groups. Furthermore, genotyping results based on assays from the signatures provided here are easily transferred between institutions, readily interpreted phylogenetically and simple to adapt to new genotyping technologies.

## Introduction

Sequence based DNA signatures are widely used for molecular typing as they provide unambiguous results that are easily transferred and compared between labs. In this era of rapid and inexpensive sequencing, whole genome sequence comparisons often reveal many polymorphisms that can be used to develop new assays for increased discrimination among samples and to better define phylogenetic relatedness. Despite drastic reductions in cost, whole genome sequencing is still expensive relative to other typing technologies. As well, the data handling, processing, and interpretation required for whole genome sequence analyses make sub-genome typing methods more viable when many samples need processing. For phylogenetic and population genetic inferences, a large sample size is also important as samples are compared to each other and accuracy of conclusions is directly tied to comprehensive sampling. Unfortunately, switching to new typing methods often results in lost information between old and new systems as data cannot be directly compared. As such, past data and efforts may be simply discarded or, when possible, old samples may be re-analyzed with the new typing scheme (for example, see [1]). Ideally, new signatures or assays should not only be transferrable between labs, but also enable newly typed samples to be directly compared to existing collections. For *Coxiella burnetii*, it is particularly important to compare typing results to other collections as *C. burnetii* collections are rare, sparse and not easily transferred due to select agent regulations and biosecurity concerns. In order to better understand epidemiological patterns we have therefore built upon an existing sequence based typing scheme to produce a few simple and rapid assays whose results are unambiguous, easily transferrable, and can be directly compared to the largest characterized collection of *C. burnetii* in the world.

*C. burnetii* causes the zoonotic disease Q fever [2]. It is prevalent throughout the world and infects many hosts, including ticks, livestock, wild animals, and humans [3]. Because symptoms are often flu-like and the disease is typically self-limiting, Q fever is likely under-diagnosed in most countries. In <1% of human cases infection can become chronic, often leading to endocarditis, and in some cases, death [4]. The low infectious dose (1–10 bacteria), aerosol route of infection, and extraordinary resistance to environmental stressors of *C. burnetii* results in the potential for rapid long-distance dispersal and its classification as a CDC category B bioterrorism agent (http://www.selectagents.gov/select%20agents%20and%20Toxins%20list.html) [5].

Despite the serious nature of Q fever, little is known about the prevalence and dissemination patterns of *C. burnetii.* Most genotyping methods are cumbersome and require relatively large quantities of DNA. Before the very recent development of a cell-free growth procedure [6], propagation required cell tissue culture or proliferation in embryonated eggs. Even with this significant improvement, culturing still requires a select agent facility, considerable expertise, and is a slow process. Thus, in the rare instances where a case of Q fever is identified, it is not likely that a sample will be successfully cultured and genotyped. Therefore, tools that facilitate the comparison of isolates or field-collected strains are particularly important.

The most diverse published collection of *C. burnetii* is maintained by the Rickettsial Unit in Marseille, France. As of February, 2011, this publicly available database (http://ifr48.timone.univ-mrs.fr/MST_Coxiella/mst) listed 170 samples that have been genotyped using multispacer sequence typing (MST) which involves sequencing ten intergenic regions for a total sequence length of ∼4,813 bp [7]. These regions exhibit single nucleotide polymorphisms (SNPs) and single nucleotide and multiple nucleotide insertions or deletions (indels) which result in 34 genotypes. Typing schemes using multiple locus VNTR (variable number tandem repeats) analysis (MLVA) have also been developed for *C. burnetii* [8], [9] and provide increased resolution by way of 36 genotypes among 42 samples [8]. Unfortunately, comparisons of phylogenetic results obtained from the two typing methods are not straightforward although general groupings can be compared if some isolates are analyzed with both methods. Also, VNTR results across labs are difficult to compare as equipment may differ and some degree of variation can be expected between runs in the same laboratory. The Marseille collection and MST results therefore offer a particularly valuable resource for understanding the genetic diversity, relatedness and geographic distribution of *C. burnetii*. As such, this collection represents a foundation that can be built upon by typing other collections in a manner where results can be directly compared. This will ultimately increase the size and geographic distribution of samples available for epidemiologic analyses. Here, we aim to exploit past genotyping efforts by extracting SNP signatures from MST loci and targeting them using rapid and inexpensive SNP assays. These SNP data allow us to assign isolates to previously described genomic groups [10] and MST genotypes [7] and thus compare additional isolates or strains to established datasets.

## Methods

### Assessment of MST loci

We further analyzed the phylogenetic results reported by Glazunova et al. [7] to assess which, if any polymorphisms could be extracted as stand-alone signatures that could define genotypes and genomic groups [7], [10]. We were particularly interested in SNPs as they are most likely to be most evolutionarily stable and thus yield accurate phylogenies [11]. Importantly, well designed SNP based assays are less cumbersome, less expensive, and more amenable to low quality and/or quantity DNA than sequencing.

Sequences for all MST alleles were downloaded (http://ifr48.timone.univ-mrs.fr/MST_Coxiella/mst/group_detail) and for each locus alignments of alleles were made using the Clustal W alignment algorithm in MegAlign (DNAStar, Madison, WI). We also determined the MST allele sequences *in silico* for the seven *C. burnetii* whole genome sequences available from NCBI (http://www.ncbi.nlm.nih.gov/genomeprj/16724). These alleles were blasted against the MST database and novel allele sequences were added to the alignments described above. We used these alignments (Fig. S1) to identify all polymorphisms and categorize them as indels, tri-allelic SNPs, or bi-allelic SNPs (Table S1).

**Figure 1:**
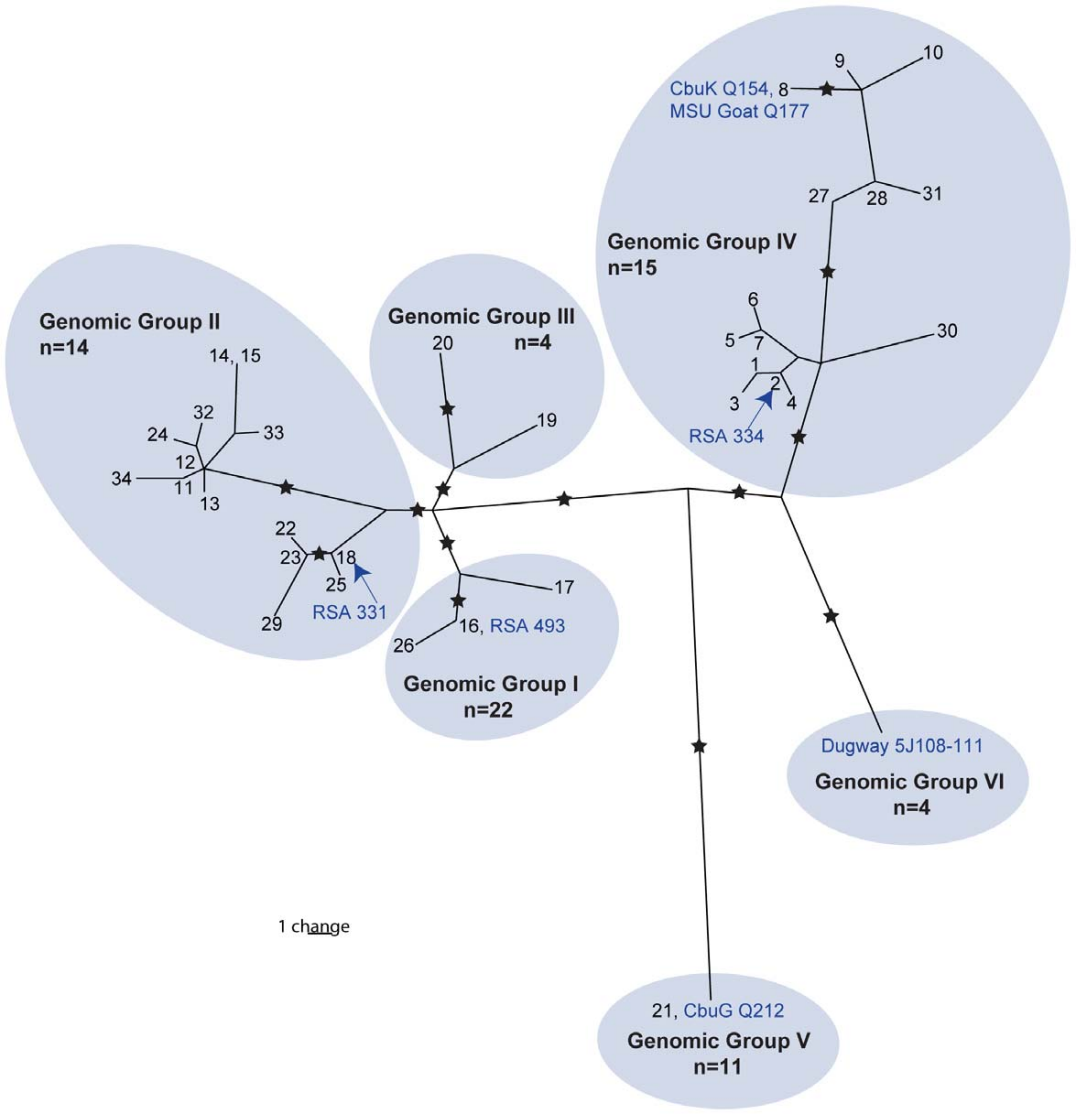
Maximum parsimony phylogeny of 35 MST genotypes for *Coxiella burnetii*. This phylogenetic tree has a homoplasy index of 0.0909 and was drawn as described in the methods and results using the 112 polymorphisms listed in Table S1. The 34 MST genotypes and their positions on the phylogeny are given along with a novel MST genotype derived from *in silico* analysis of the whole genome sequence Dugway 5J108–111. The remaining six whole genome sequences are shown in blue text alongside their corresponding MST genotype as determined by *in silico* analysis, however analyses of MSU Goat and African Q revealed alleles at only 9 of 10 loci, therefore they are assigned to their most likely MST genotype. Our alignments showed no differences between MST genotypes 14 and 15. Stars indicate the 14 branches that were targeted for assay development. Our predicted genomic groups based on Hendrix et al. [10] are highlighted along with the total number of samples (n) from our study that genotyped into these groups.

We drew a maximum parsimony tree (Fig. 1 and Fig. S2) with PAUP* 4.0b [12] using all polymorphic characters. As homoplasy (shared alleles not due to descent) levels are low, we report the homoplasy index as a more appropriate indication of accuracy than bootstrapping [13]. As no evidence of lateral gene transfer has been reported for *C. burnetii*, we expected phylogenetic patterns to reflect a completely clonal mode of inheritance and thus show little homoplasy. We mapped all characters onto the tree and were thus able to identify the phylogenetic location of all characters as well as those which were homoplastic (Table S1 and Fig. S2).

### SNP selection and assay development

We developed genotyping assays based on 14 SNPs. Twelve SNPs that define the major clades were used to develop Melt-MAMA assays (Table 1) as described by Vogler et al. [14]. Briefly, the melt-MAMA design utilizes allele-specific mismatch amplification mutation assay primers [15] coupled with GC- or T-rich primer tails. These tails force allele specific melt properties for PCR amplicons, allowing allelic differentiation via melt curve analysis. Two other SNPs from MST allele comparisons were used to develop TaqMan minor groove binding dual-probe assays according to Easterday et al. [16] (Table 2) and to illustrate that multiple SNP-interrogation methods can be used to assay SNP signatures.

**Table 1:** Assay information for the 12 Melt-MAMA assays used in this study.

Branch^a^	Assay name^b^	SNP position in RSA493 (GenBank: AE016828.2)	Base call RSA493/alternate^c^	Melt-MAMA primer^d^	Melt-MAMA primer sequences 5′→3′^e^	Primer conc. (nM)	Number of PCR cycles	Approximate T_m_ (°C)^f^
Br.I.001	Cox18bp376	283,398	T/C^h^	RSA493	cggggcggggcggggcggggCAGCGCCTCCCTTTTTTcA	1125	33	81.0
				Alt	ttttttttttttttttttttAGCGCCTCCCTTTTTTcG	225		76.0
				C	GCTTAAGTTGGCGCTTCTGTG	300		
Br.I.003	Cox51bp356	824,910	A/C^h^	RSA493	cggggcggggcggggcggggGTATCTGCTAAAAAGCTAGCGAAAcT	150	40	80.0
				Alt	ttttttttttttttttttttGTATCTGCTAAAAAGCTAGCGAAAgG	750		76.0
				C	GACTTTATCATCGCCCGGTAG	300		
Br.IV.001	Cox18bp34	283,056	A/G^h^	RSA493	tttttttttttttttGCTTTAAATTTTTGATAGGGGTATAACTAaT	750	33	74.5
				Alt	cggggcggggcggggCTTTAAATTTTTGATAGGGGTATAACTAgC	150		79.0
				C	CGAATTAGCCAATCGTGGC	300		
Br.III.001	Cox5bp109	77,615	T/C	RSA493	cggggcgggttttttttttttttttttttTGATATGCTTAACATAAGCACGTATTcT	300	40	75.0
				Alt	cggggcggggcggggcggggGATATGCTTAACATAAGCACGTATTcC	150		80.0
				C	CTCTCCTTAACCCTCTCCTCGA	300		
Br.IV.011	Cox22bp118	378,789	C/A	RSA493	tttttttttttttttttttttttttGTGCGGAGAAAATATTGAACGtC	150	33	73.7
				Alt	cggggcggggcggggcggggcggggGCGGAGAAAATATTGAACGgA	150		76.9
				C	CGCTAAGCAAAAAGTGAGTGATAGC	300		
Br.II.007	Cox51bp492	825,046	G/A	RSA493	cggggtttttttttttttttTCAATTTTTCAAGCGGCATAaG	150	33	74.0
				Alt	cggggcggggcggggTTTCAATTTTTCAAGCGGCATAtA	150		78.0
				C	GACGGGATAAGTCGGGAGG	300		
Br.IV/VI	Cox57bp327	893,096	G/A	RSA493	ggggttttttttGATAACAAGCTTTATTTGCCGACTaG	875	30	72.3
				Alt	cggggcggggcgggggATAACAAGCTTTATTTGCCGACTcA	175		75.0
				C	cgccccgATCAGTTAGTCAGATATCTTTAATTTTAATCGG	300		
Br.III.003	Cox56bp10	886,387	A/G^h^	RSA493	tttttttttttttttATAGTCTTAGCTCTGATTGCAACAaT	750	33	75.3
				Alt	cggggcggggcggggATAGTCTTAGCTCTGATTGCAACAtC	150		80.9
				C	CAAGCTCTCTGTGCCCAAT	300		
Br.II.004	Cox37bp215	657,611	C/T^h^	RSA493	cgcgtttttttttttttttCCCCTCTTTAAGTTTATCTGGGtG	150	33	75.3
				Alt	cggggcggggcggggCCCTCTTTAAGTTTATCTGGGcA	150		78.5
				C	cgggcgggTCTGGTTAAACCTTTCAAGGAGG	300		
Br.IV.015	Cox51bp67	824,623	T/C	RSA493	ttttttttttttttttttttGTTGAGAGAATAGTGGGTTTTACTAATgTT	150	35	70.0
				Alt	cggggcggggcggggcggggTGAGAGAATAGTGGGTTTTACTAATTgC	150		77.3
				C	CGGGCACTCACAATTACTTAAATTATCGTACGAA	300		
Br.VI.001	Cox20bp155	365,416	G/A	RSA493	tttttttttttttttGGTATTCAAGGCTTGTGAGATAAACtG	750	40	73.0
				Alt	cggggcggggccgggGTATTCAAGGCTTGTGAGATAAACcA	150		78.6
				C	CAGGTTCAGCGATTGCATTAG	300		
Br.II.001	Cox18bp166^g^	283,188	C/G^h^	RSA493	ttttttttttttttttttttTTGAAGCATACAAAACCTTCAtGG	150	33	77.4
				Alt	cggggcggggcggggcggggTTGAAGCATACAAAACCTTCACtC	750		81.3
				C	cgggcgggGAGCGAGGTAAAGAGGCAATATC	300		

a Branch targeted on the phylogenetic tree (see Fig. S2).

b Assay name given as the MST locus [7] containing the SNP target of interest followed by the base position of the SNP within the allele alignments for that locus (see also Fig. S1).

c Base call in the whole genome sequence of Nine Mile phase I (RSA 493) is listed first, followed by the alternate allele.

d RSA493, melt-MAMA primer that is specific to the Nine Mile phase I (RSA 493) allele; Alt, melt-MAMA primer specific to the alternate allele; C, consensus primer.

e Primer tails that do not match the native sequence along with the penultimate or antepenultimate mismatch bases are shown in lower case.

f T_m_ can vary based on the instrument used, typically ±1°C.

g Assay that had a single incidence of homoplasy for one sample (see text).

h Assays designed on the reverse compliment.

**Table 2:** Assay information for the two TaqMan minor-groove binding dual-probe assays used in this study.

Branch^a^	Assay name^b^	SNP position in RSA493 (GenBank: AE016828.2)	Base call RSA493/alternate^c^	Primer/Probe Name^d^	TaqMan Primer/probe sequences 5′→3′^e^
Br.V.001	Cox5bp81	77,587	G/C	Forward	CGAGGTGTTTGGTGTGTTGAA
				Reverse	GGAGAGGGACAATACGTGCTTATG
				RSA493	6FAM-TTCGCAgTGATATGC-MGB
				Alt	VIC-CTAGTAATTTCGCAcTGATATGC-MGB
Br.I/II/III	Cox22bp91	378,762	T/C	Forward	GGTGAATAGATTACGCCTTCCATT
				Reverse	CGCCTTATGTAATTGTCGTTCAAT
				RSA493	6FAM-TGGTGCTCCCtTGTA-MGB
				Alt	VIC-TGCTCCCcTGTAGTGC-MGB

a Branch targeted on the phylogenetic tree (see Fig. S2).

b Assay name given as the MST locus [7] containing the SNP target of interest followed by the base position of the SNP within the allele alignments for that locus (see also Fig. S1).

c Base call in the whole genome sequence of Nine Mile phase I (RSA493) is listed first, followed by the alternate allele.

d RSA493, TaqMan-MGB probe that is specific to the Nine Mile phase I (RSA493) allele; Alt, TaqMan-MGB probe that is specific to the alternate allele.

e Lower case nucleotides in the probe sequences indicates the position and base of the target SNP; probe sequences also show the fluorescent dye label on the 5′ end and the minor groove binder on the 3′ end.

### Bacterial strains and genotyping

*Coxiella burnetii* isolates used in this study and their associated epidemiological data are listed in Table S2. As 18 of our 63 isolates overlapped with isolates used by Glazunova et al. [7], we were able to compare and evaluate the consistency of the results. Additionally, 21 of our isolates overlapped with those used by Hendrix et al. [10] who describe genomic groups defined by restriction enzyme banding patterns. This overlap allowed us to predict genomic groups based on our phylogeny.

Genomic DNA was isolated using the QiaAmp DNA Mini Kit (Qiagen, Valencia, CA, USA), following the tissue lysis protocol with proteinase K lysis performed at 56°C overnight. For the 12 melt-MAMA assays, 1 µL of DNA was used in a total PCR reaction volume of 10 µL that contained 1× SYBR® Green PCR Master Mix (Applied Biosystems by Life Technologies, Foster City, CA, USA), 300 nM consensus primer, and variable amounts of allele-specific primers (see Table 1). Thermal cycling conditions were: 50°C for 2 min., 95°C for 10 min., followed by the specified number of cycles (see Table 1) of 95°C for 15 sec., 55°C for 1 min. and concluding with a dissociation stage of 95°C for 15 sec., 55°C for 15 sec., 95°C for 15 sec. Analysis of melt curves were performed as described by Vogler et al. [14]. For select samples from each genogroup, results obtained by Melt-MAMA assays were confirmed by MST of entire loci as described by Glazunova et al. [7] with the exclusion of using plasmid vectors for cloning and amplification.

For the two TaqMan minor-groove binding dual-probe assays, 1 µL of DNA was also used in a total reaction volume of 10 µL that contained 1× TaqMan® Genotyping Master Mix (Applied Biosystems by Life Technologies, Foster City, CA, USA), 900 nM of each primer and 200 nM of each probe (Table 2). Thermal cycling conditions were: 50°C for 2 min., 95°C for 10 min., followed by 40 cycles of 95°C for 15 sec., 60°C for 1 min. Results were analyzed as described by Easterday et al. [16]. All assays were run on an Applied Biosystems 7900HT Fast real-time PCR system with SDS v2.3 or v2.4 software. MST genotype designation(s) and phylogenetic group predictions were based on the results from the 14 SNP assays.

## Results

### Assessment of MST loci

Mapping the phylogenetic location of all polymorphisms on a parsimony tree allows for the choosing of specific characters on particular branches to be used in defining a clade or genotype with little likelihood that unrelated isolates will share alleles. Maximum parsimony analysis of polymorphic characters from MST data resulted in 16 equally parsimonious trees with a homoplasy index of 0.0909 indicating that most loci have only mutated once over the evolutionary history of the species. The 16 trees differed from each other by minor topological changes and branch length variations (data not shown). A subsequent maximum parsimony phylogenetic analysis of only the non-homoplastic SNP loci produced a single tree on which the homoplastic SNPs and all indels were added using maximum parsimony criteria (Fig. 1). This tree had the same topology as 4 of the 16 trees that were initially created and was largely congruent with the tree published by Glazunova et al. [7], however we used updated data (http://ifr48.timone.univ-mrs.fr/MST_Coxiella/mst), included a novel sequence type (ST), and used a parsimony approach rather than UPGMA. There were 91 SNPs (eight of which were homoplastic) and 21 indels (four of which were homoplastic) (Table S1). We were able to determine the MST genotypes of the seven available whole genome sequences (Fig. 1, Table S2). For four of these genomes (RSA 493, RSA 331, CbuG Q212, CbuK Q154) the MST genotypes were comprised of combinations of already published alleles from Glazunova et al. [7] and resulted in previously described genotypes (genotypes 16, 18, 21, and 8 respectively) therefore, the *in silico* sequence data for these genomes are not shown. Analyses of the two shotgun genomes, MSU Goat (Q177; GenBank: AAUP00000000.2) and African Q (RSA 334; GenBank: AAYJ00000000.1) also revealed previously published alleles at all loci except Cox56 which is absent from these genomes. For these two genomes, the combination of known alleles at the 9 present loci directly matched a single genotype in the Marseille database each, suggesting that these samples are most likely genotypes 8 (MSU Goat) and 2 (African Q), or at most, single-locus variants of these genotypes (Fig. 1). Finally, *in silico* analysis of the Dugway 5J108–111 genome (GenBank: CP000733) revealed new alleles at 8/10 MST loci and thus created a new branch on the tree (Fig. 1) not previously described in Glazunova et al. [7]. These new allele sequences are shown in the alignments in Fig. S1. The 14 phylogenetic branches selected for assay development are also shown in Figure 1.

### Genotyping

For assay development, we selected 14 SNPs that were likely to provide discrimination amongst the major genomic groups described by Hendrix et al. [10] with some resolution within these groups (Fig. 1). Because of their phylogenetic positions, our assays were not expected to provide resolution down to a single MST genotype in all cases. However, we were able to assign 25/63 isolates to a single MST genotype while all other isolates (with the exception of Deer Q) could be placed in a single genomic group containing >1 genotype (Table S2). We list additional signatures that can be used to provide increased resolution in most groups (Table S1 and Figure S2).

Our SNP data are mostly congruent with expected genotypes (Table S2). In the eight instances where the isolates from our collection matched whole genome sequences, *in silico* SNP analysis of these 14 markers matched SNP calls determined using our assays for 7/8 samples. The exception was the isolate African Q (RSA 334). *In silico* analysis narrowed this sample to MST genotypes 1–7 or 30 (genomic group IV) whereas laboratory analysis placed this sample in MST genotypes 16 or 26 (genomic group I) with differences at 5/14 assays (Fig. 1, Table S2). Due to the evolutionary stable nature of SNPs, the number of assays tested, and the number of differences, the isolate used for whole genome sequencing is not likely from the same stock as the isolate analyzed here. Indeed, despite their matching names, these isolates are only distantly related.

Two other genotype results are noteworthy. First, there was one incidence of homoplasy in our dataset (Table S2). The Cox18bp166 assay gave an unexpected result (based on the 13 other assays) for isolate L 35. In conjunction, the 13 other assays suggested that this sample is genotype 16 or 26 while the Cox18bp166 was in disagreement and suggested it was in an entirely different clade. Second, one isolate (Deer Q) produced mixed alleles at 4 loci. We were able to determine that this sample contained genotype 16 (or 26) mixed with genotype 21 (see Table S2).

### Collection comparisons

Many genotypes appear to have a wide geographic distribution. In our study, the greatest number of isolates was assigned to the genotype 16 and 26 group. This group also appears to be one of the more geographically diverse as epidemiological data lists sample origins from Africa, Australia, Cyprus, Panama, Scotland, Slovak Republic, and various states in the USA. This geographic diversity also appears to be true for samples of these same genotypes in Glazunova et al. [7] which includes isolates from Africa, USA, France, Romania, Slovak Republic, Germany, Japan and Uzbekistan. In contrast to this are samples determined to be genotype 8. In this study, most samples in this group (n = 8) were from the USA, suggesting a more localized distribution of this genotype. However, Glazunova et al. [7] includes 28 samples in genotype 8, twenty-four collected in France and the remaining four from the USA and Spain, suggesting a more widespread distribution of this genotype. Perhaps a more interesting aspect of this genotype is that it is associated with chronic disease in humans, and the only animal species it has been collected from is goats. The 28 samples in genotype 8 in Glazunova et al. [7] contain 25 from chronic human infections (endocarditis or aneurism), one from a human abortion, and two from goat abortions. In the current study, samples in genotype 8 include four from chronic human endocarditis (heart valves), four from goat tissue from aborted kids, and a single environmental sample from a farm in California, USA, with goats. The power of direct comparisons among collections is also illustrated in samples assigned to genotype 20. Taken alone, each study may suggest that genotype 20 has a primary geographic distribution in a particular region (France versus USA), however, as both studies show multiple contemporary isolates from this genotype, this is clearly not the case.

In this study, we found a new genotype via *in silico* analysis of the whole genome sequence Dugway 5J108–111. This was unexpected because an isolate with a matching name was included in the study by Glazunova et al. [7] and assigned to a very distant genotype (genotype 20). The lack of homoplasy at any of the characters used to place this genome on the phylogeny suggests that this phylogenetic placement is not a result of genome sequencing errors. Furthermore, the isolates tested here from the collection at the Centers for Disease Control and Prevention (CDC) in Atlanta had three other samples obtained from rodents in Utah that matched our Dugway 5J108–111 *in silico* genotype, suggesting that this truly is a new genotype. This is also supported by the genomic grouping for two of these isolates in Hendrix et al. [10]. Interestingly, a fifth sample from Utah obtained from a tick did not genotype as the other 4 Dugway strains, but instead matched genotypes in the diverse 16 and 26 genotype group.

We found another instance of a strain with a matching name from Glazunova et al. [7], but not a matching genotype. In our study, the isolate named “Florian” was determined to be genotype 22, 23, or 29 (Table S2). However Glazunova et al. [7] determined their sample to be genotype 18. These genotypes are closely related to each other (Fig. 1) and only a single SNP difference at locus Cox37bp215 differentiates genotype 18 from the group containing genotypes 22, 23, and 29. To confirm our results, we sequenced the Cox37 spacer for the Florian sample and confirmed that the nucleotide at this position was an A (data not shown), indicating that our isolate is indeed from the group containing genotypes 22, 23, or 29. The passage history of the Florian isolate in each of the two laboratories from which the samples were derived is not known. It is possible that extensive propagation of the isolate in one laboratory or the other may have led to the single SNP change that was found.

## Discussion

The release of the several whole genome sequences of *C. burnetii* isolates [17] has facilitated the development of genotyping schemes and the characterization of collections. Due to the complexity of culturing *C. burnetii* and select agent restrictions, collections of *C. burnetii* are small and rare making it all the more important to build upon existing work in order to facilitate inter-laboratory comparisons among the collections that are available. Such comparisons will lead to a better understanding of the phylogeographic distribution of this pathogen historically, at present, and in the future.

In this study, we exploit SNP signatures from the readily available MST scheme for *C. burnetii* [[7]; http://ifr48.timone.univ-mrs.fr/MST_Coxiella/mst/group_detail] and convert them into inexpensive, high-throughput, transferrable assays that can be used to quickly determine the genomic group and MST genotype of existing samples. This study further adds 41 isolates from a large collection of *C. burnetii* maintained in the United States to the total number of strains with MST information, thereby expanding the data available for worldwide comparisons while demonstrating proof of principle of our methods. This will hopefully encourage further genotyping that builds upon our understanding of the phylogeography of *C. burnetii* as new isolates are collected.

The strength of the typing scheme presented is that it allows for accurate identification of genotypes and rapid characterization of new isolates or field-collected samples from natural outbreaks or from a suspected intentional release. The CDC will be evaluating the use of the method for such purposes and for forensic investigations, association of particular strains with virulence, reservoir specificity, and the geographic origin of strains. An example of this use is shown in the analysis of genotype 8 which appears to be associated with chronic human disease, particularly Q fever endocarditis, with goats as the reservoir. It may be prudent to evaluate acute humans infections associated with contact with goats and goat farming products to determine if genotype 8 strains are involved. Human infections identified as being due to genotype 8 strains may warrant more intense scrutiny and follow up evaluation due to the potential for development of chronic disease. While the treatment regimen for all strains of *C. burnetii* is identical, it may be appropriate to emphasize the treatment of acute illness caused by certain strains associated with chronic infections. However, of particular interest is that none of the 35 genotype 8 isolates have yet been associated with acute human infection suggesting that these isolates may cause a benign or asymptomatic acute infection that is therefore generally not treated and subsequently may develop into chronic disease such as endocarditis. Additional studies, and the analysis of difficult to identify asymptomatic acute infections, will be necessary to determine the full pathogenic potential of the genotype 8 strains.

While we provide the signatures that will discriminate among all MST genotypes, we did not develop all such signatures into assays. There are 44 phylogenetic branches supported by SNPs, yet we only designed assays for SNPs on 14 branches. We selected the major branches for assay development along with a small number of other branches that would narrow down the list of possible genotypes within a genomic group for the bacterial strains tested. To do this, we designed and tested assays in an iterative fashion. As we did not have access to samples from each MST genotype, we did not design assays for each one as we would not be able to thoroughly test such assays. The signatures for every branch on the tree however are listed in Table S1 and could readily be developed into assays if needed. Also, some differences among MST genotypes are solely due to indels and were therefore not incorporated into assays that could discriminate between such genotypes.

While rapidity and simplicity are important advantages of SNP based genotyping assays, another benefit is robustness. As SNP mutation rates are low, the likelihood of convergent or reverse mutations are low, making homoplasy unlikely in the absence of selection. Furthermore, homoplastic data are not likely to result in incorrect phylogenetic placement as the phylogenetic signal will conflict with a congruent signal produced by other loci. This redundancy can occur even if a single locus is selected to represent each branch (canonical SNP). Of the 63 strains and 7 whole genome sequences genotyped against 14 SNP loci, there was only a single instance of homoplasy (1/972 data points), confirming the evolutionary stability of these signatures. As SNPs from multiple branches are assayed against each isolate, this provides a level of redundancy that makes it easy to spot suspicious results that arise if two assays on different parts of the phylogeny place the same isolate in two exclusive clades, as was seen with sample L 35 and assay Cox18bp166.

Of note is that the typing scheme presented here is designed to be fully comparable with MST genotype data and not to identify novel genotypes. However, it is reasonable to assume that novel genotypes may be found. For example, *in silico* analysis of the whole genome sequence of Dugway 5J108–111 in this work revealed novel alleles at 8/10 loci, resulting in a novel genotype, but also see Mediannikov et al. [18] where isolates from ticks resulted in new genotypes that were comprised of different combinations of already known alleles rather than new alleles.

The typing scheme presented here is compatible with current as well as emerging genotyping technologies. SNP based assays are highly amenable to adaptation to different platforms [19], [20], chemistries (TaqMan, SYBR), and a variety of allelic detection machinery [16], [21]. For example, TaqMan assays have the potential for extremely sensitive detection and have been shown to successfully genotype single molecules [16]. Such sensitivity means that these assays can be used to genotype samples collected from the environment without the need for culturing. As these assays are all sequence based, they are also compatible with whole genome sequencing and, unlike VNTR assays, will not likely be sensitive to different sequencing platforms. Analysis of whole genome sequence data will reveal alleles at all MST loci, allowing rapid placement of a genome onto the MST phylogeny. As whole genome comparisons will reveal more pairwise polymorphisms than MST, if the sequenced genomes are from the same MST genotype, SNPs might be found that can be developed into SNP based assays for testing against other samples within that genotype for added resolution.

In summary, while whole genome sequencing of every sample is currently impractical, the method we describe here can serve as a bridge between conventional PCR based genotyping and whole genome sequencing. We have developed 14 assays whose data can be used to place *C. burnetii* isolates into the phylogenetic context of six genomic groups and 35 MST genotypes, allowing for comparison of existing and new isolates. As these are sequence based signatures, data collected using these assays will remain useful even as platforms and technologies change and can be queried using *in silico* methods as more *C. burnetii* isolates are whole genome sequenced.

## Supporting Information

Figure S1 **Alignments of MST alleles.** Clustal W alignments of all MST alleles per locus, including *in silico* derived alleles from the whole genome sequence of Dugway 5J108–111 (CP000733.1). To the left of each alignment, the allele names from Glazunova et al. [7] are shown as the MST spacer name, followed by the allele number (for example: Cox2.1 denotes the sequence from spacer Cox2, allele number one). Alleles derived from *in silico* analysis of Dugway 5J108–111 are listed as MST spacer name followed by Dugway_5J108–111 (example: Cox2.Dugway_5J108–111). With the exception of the Cox22 and Cox37 alleles, all alleles from Dugway 5J108–111 were novel; Cox22 matched allele 6, Cox37 matched allele 4. Nucleotide position per allele is shown above the alignments. Shaded regions indicate areas of identity; unshaded regions denote polymorphisms and have either a dot if the nucleotide matches that of the base found in allele 1 or the polymorphic base call: A, C, G, T or a dash to denote a deletion.
(PDF)

Figure S2 **Phylogenetic tree of MST genotypes with labeled branches.** Panel A: complete tree as in Figure 1. Panels B–C provide an expanded view of different groups to better visualize branch names; branch names were assigned based on the genomic group nomenclature (I–VI) described in Hendrix et al. [10] and can be used with Table S1 to determine the location of each of the 112 loci on this tree.
(PDF)

Table S1 **Phylogenetic characterization of the 112 polymorphisms from MST sequence comparisons.** *^a^*Locus name is given as the MST locus followed by the base position in the alignment (Fig. S1). *^b^*Branch indicates the phylogenetic location(s) of each polymorphism on the phylogenetic tree (Fig. S2). *^c^*Locus type describes the nature of the polymorphism: bi-allelic SNP, tri-allelic SNP or indel; autapomorphies are also indicated. *^d^*This locus was not homoplastic based on *in silico* analyses but was found to be homoplastic for a single sample when assayed against the entire panel of DNA (see text). *^e^*Assays presented in this work.
(XLS)

Table S2 **Epidemiological and results data for samples used in this study.** *^a^*Expected MST genotype based on overlap of samples with Glazunova et al. [7] (denoted with *^b^*) or from *in silico* analysis of whole genome sequences from this work (denoted with *^c^*). Samples with a listing of n/a were not reported in Glazunova et al. [7] (based on a comparison of sample name alone) nor had whole genome sequence available for *in silico* analysis. Thus we could not predict the genotype based on other studies. *^d^*Observed MST genotype(s) based on the results from 14 SNP assays presented here. *^e^*Vaccine strain. *^f^*Sample contained mixed genotypes as determined by both alleles amplifying at four loci. *^g^*Allele determined to be homoplastic based on allele calls at the 13 other loci. Abbreviations and symbols: WGS, Whole genome sequences where sequences and epidemiological data were used from http://www.ncbi.nlm.nih.gov and SNP data was derived using *in silico* techniques; EP, egg passage; -, epidemiological data is missing or unknown; ?, alleles were absent when genotyped using *in silico* methods; u, data was undetermined; nt, assay was not tested.
(XLSX)

## References

[1] Huijsmans CJ, Schellekens JJ, Wever PC, Toman R, Savelkoul PH (2011). SNP-genotyping of a *Coxiella burnetii* outbreak in the Netherlands.. Appl Environ Microbiol.

[2] Derrick EH (1937). “Q” Fever, A New Fever Entity: Clinical Features, Diagnosis and Laboratory Investigations.. The Medical Journal of Australia.

[3] Waag DM (2007). Q Fever.. Medical Aspects of Biological Warfare: Department of Defense, Office of the Surgeon General, US Army, Borden Institute..

[4] Schimmer B, Morroy G, Dijkstra F, Schneeberger PM, Weers-Pothoff G (2008). Large ongoing Q fever outbreak in the south of The Netherlands, 2008.. Euro Surveill.

[5] McQuiston JH, Childs JE (2002). Q fever in humans and animals in the United States.. Vector Borne Zoonotic Dis.

[6] Omsland A, Cockrell DC, Howe D, Fischer ER, Virtaneva K (2009). Host cell-free growth of the Q fever bacterium *Coxiella burnetii*.. Proc Natl Acad Sci U S A.

[7] Glazunova O, Roux V, Freylikman O, Sekeyova Z, Fournous G (2005). *Coxiella burnetii* genotyping.. Emerg Infect Dis.

[8] Arricau-Bouvery N, Hauck Y, Bejaoui A, Frangoulidis D, Bodier CC (2006). Molecular characterization of *Coxiella burnetii* isolates by infrequent restriction site-PCR and MLVA typing.. BMC Microbiol.

[9] Svraka S, Toman R, Skultety L, Slaba K, Homan WL (2006). Establishment of a genotyping scheme for *Coxiella burnetii*.. FEMS Microbiol Lett.

[10] Hendrix LR, Samuel JE, Mallavia LP (1991). Differentiation of *Coxiella burnetii* isolates by analysis of restriction-endonuclease-digested DNA separated by SDS-PAGE.. J Gen Microbiol.

[11] Pearson T, Okinaka RT, Foster JT, Keim P (2009). Phylogenetic understanding of clonal populations in an era of whole genome sequencing.. Infect Genet Evol.

[12] Swofford DL (2003). PAUP*. Phylogenetic Analysis Using Parsimony (*and Other Methods)..

[13] Archie JW, Sanderson MJ, Hufford L (1996). Measures of homoplasy.. Homoplasy: The recurrence of similarity in evolution.

[14] Vogler AJ, Birdsell D, Price LB, Bowers JR, Beckstrom-Sternberg SM (2009). Phylogeography of *Francisella tularensis*: global expansion of a highly fit clone.. J Bacteriol.

[15] Cha RS, Zarbl H, Keohavong P, Thilly WG (1992). Mismatch amplification mutation assay (MAMA): application to the c-H-ras gene.. PCR Methods Appl.

[16] Easterday WR, Van Ert MN, Zanecki S, Keim P (2005). Specific detection of *Bacillus anthracis* using a TaqMan mismatch amplification mutation assay.. Biotechniques.

[17] Seshadri R, Paulsen IT, Eisen JA, Read TD, Nelson KE (2003). Complete genome sequence of the Q-fever pathogen *Coxiella burnetii*.. Proc Natl Acad Sci U S A.

[18] Mediannikov O, Fenollar F, Socolovschi C, Diatta G, Bassene H (2010). *Coxiella burnetii* in humans and ticks in rural Senegal.. PLoS Negl Trop Dis.

[19] Gaudet M, Fara AG, Beritognolo I, Sabatti M (2009). Allele-specific PCR in SNP genotyping.. Methods Mol Biol.

[20] Van Ert MN, Easterday WR, Simonson TS, U'Ren JM, Pearson T (2007). Strain-specific single-nucleotide polymorphism assays for the Bacillus anthracis Ames strain.. J Clin Microbiol.

[21] Cruz RE, Shokoples SE, Manage DP, Yanow SK (2010). High-throughput genotyping of single nucleotide polymorphisms in the *Plasmodium falciparum* dhfr gene by asymmetric PCR and melt-curve analysis.. J Clin Microbiol.

