# Supplementary material for: Rapid Typing of Coxiella burnetii
Source: PLoS One. 2011 Nov 2;6(11):e26201. doi: 10.1371/journal.pone.0026201 (PMC3206805; doi:10.1371/journal.pone.0026201)
Supplement: Figure S1 — Alignments of MST alleles. Clustal W alignments of all MST alleles per locus, including in silico derived alleles from the whole genome sequence of Dugway 5J108–111 (CP000733.1). To the left of each alignment, the allele names from Glazunova et al. [7] are shown as the MST spacer name, followed by the allele number (for example: Cox2.1 denotes the sequence from spacer Cox2, allele number one). Alleles derived from in silico analysis of Dugway 5J108–111 are listed as MST spacer name followed by Dugway_5J108–111 (example: Cox2.Dugway_5J108–111). With the exception of the Cox22 and Cox37 alleles, all alleles from Dugway 5J108–111 were novel; Cox22 matched allele 6, Cox37 matched allele 4. Nucleotide position per allele is shown above the alignments. Shaded regions indicate areas of identity; unshaded regions denote polymorphisms and have either a dot if the nucleotide matches that of the base found in allele 1 or the polymorphic base call: A, C, G, T or a dash to denote a deletion. (PDF) [file pone.0026201.s001.pdf]

```
                                 10         20         30         40         50         60         70         80         90        100
                         ....|....| ....|....| ....|....| ....|....| ....|....| ....|....| ....|....| ....|....| ....|....| ....|....|
COX2.1                   ATGTTTAGAA CAAAATCAGT TAAGGAAACA AATGTAAATA AAGAATTGGA TTTTGAGCCC ATTGCTTCTT TCACTAAGTA ATAATTTACT TTAAAAATTT   COX2.1
COX2.2                   .......... .......... .......... G......... .......... .......... .......... .......... .......... ..........   COX2.2
COX2.3                   .......... .......... .......... G......... .......... .......... .......... .......... .......... ..........   COX2.3
COX2.4                   .......... .......... .......... G......... .......... .......... .......... .......... .......... ..........   COX2.4
COX2.5                   .......... .......... .......... G......... .......... .......... .......... .......... .......... ..........   COX2.5
COX2.6                   .......... .......... .......... G......... .......... .........T .......... .......... .......... ..........   COX2.6
COX2.7                   .......... .......... .......... G......... .......... .......... .......... .......... .......... ..........   COX2.7
COX2.8                   .......... .......... .......... G......... .......... .......... .......... .......... .......... ..........   COX2.8
COX2.9                   .......... .......... .......... G......... .......... .......... .......... ......G... .......... ..........   COX2.9
COX2.Dugway_5J108-111    .......... .......... .......... G......... .......... .......... .......... .......... .......... ..........   COX2.Dugway_5J108-111

                                110        120        130        140        150        160        170        180        190        200
                         ....|....| ....|....| ....|....| ....|....| ....|....| ....|....| ....|....| ....|....| ....|....| ....|....|
COX2.1                   TTTATTGACC GCTTAAATAC TCTAAATAAT TTTATATTTC AGCAATTCTA ATTTTGACTC TTACAGTTCC GTTATTCCCG CGCAGGCGGG AATGACGAAC   COX2.1
COX2.2                   .......... .........G .......... .......... .......... .......... .......... .......... .......... ..........   COX2.2
COX2.3                   .......... .......... .......... .......... .......... .......... .......... ..C....... .......... ..........   COX2.3
COX2.4                   .......... .......... .......... .......... .......... .......... .......... .......... .......... ..........   COX2.4
COX2.5                   .......... .......... .......... .......... .......... .......... .......... .......... .......... ..........   COX2.5
COX2.6                   .......... .......... .......... .......... .......... .......... .......... ..C....... .......... ..........   COX2.6
COX2.7                   ...T...... .......... .......... .......... .......... .......... .......... ..C....... .......... ..........   COX2.7
COX2.8                   .......... .......... .......... .......... .......... .......... .......... .......... .......... ..........   COX2.8
COX2.9                   .......... .......... .......... .......... .......... .......... .......... .......... .......... ..........   COX2.9
COX2.Dugway_5J108-111    .......... .......... .......... .......... .......... .......... .......... .......... .......... ..........   COX2.Dugway_5J108-111

                                210        220        230        240        250        260        270        280        290        300
                         ....|....| ....|....| ....|....| ....|....| ....|....| ....|....| ....|....| ....|....| ....|....| ....|....|
COX2.1                   TTAAAATTAG AATTACTGCC TTTTTTGGTT TCTTTACATT GCAAGAAAGA TTAGATAAAA TAAATACTTA TAATTAAGGA AGATTTAACG TTTCGAAAAC   COX2.1
COX2.2                   .......... .......... .......... .......... .......... .......... .......... .......... .......... ..........   COX2.2
COX2.3                   .......... .......... .......... .......... .......... .......... .......... .......... .......... ..........   COX2.3
COX2.4                   .......... .......... .......... ......G... .......... .......... .......... .......... .......... ..........   COX2.4
COX2.5                   .......... .......... .......... .......... .......... .......... .......... .......... .......... ..........   COX2.5
COX2.6                   .......... .......... .......... .......... .......... .......... .......... .......... .......... ..........   COX2.6
COX2.7                   .......... .......... .......... .......... .......... .......... .......... .......... .......... ..........   COX2.7
COX2.8                   .......... .......... .......... .......... .......... .......G.. .......... .......... .......... ..........   COX2.8
COX2.9                   .......... .......... .......... .......... .......... .......G.. .......... .......... .......... ..........   COX2.9
COX2.Dugway_5J108-111    .......... .......... .......... .......... .......... .......... .......... .......... .......... ..........   COX2.Dugway_5J108-111

                                310        320        330        340        350        360
                         ....|....| ....|....| ....|....| ....|....| ....|....| ....|....| ....|
COX2.1                   CTAGAAAGGC GAGCAGCCAT GGGAACGAAT CAA------- --ACGAAACA GAGAGAAACT CAACC   COX2.1
COX2.2                   .......... .......... .......... ...------- --........ .......... .....   COX2.2
COX2.3                   .......... .......... .......... ...------- --........ .......... .....   COX2.3
COX2.4                   .......... .......... .......... ...ACGAATC AA........ .......... .....   COX2.4
COX2.5                   .......... .......... .......... ...------- --........ .......... .....   COX2.5
COX2.6                   .......... .......... .......... ...------- --........ .......... .....   COX2.6
COX2.7                   .......... .......... .......... ...------- --........ .......... .....   COX2.7
COX2.8                   .......... .......... .......... ...------- --........ .......... .....   COX2.8
COX2.9                   .......... .......... .......... ...------- --........ .......... .....   COX2.9
COX2.Dugway_5J108-111    .......... .......... ....------ ---------- --........ .......... .....   COX2.Dugway_5J108-111
```

```
                              10         20         30         40         50         60         70         80         90        100
COX5.1                AAAACGCTCT CCGAGGTGTT TGGTGTGTTG AATAAATATT TTAGATAATG ATGATGTGAG TTTGTTCTAG TAATTTCGCA CTGATATGCT TAACATAAGC  COX5.1
COX5.2                .......... .......... .......... .......... .......... .......... .......... .......... G......... ..........  COX5.2
COX5.3                .......... .......... .......... .......... .......... .......... .......... .......... G......... ..........  COX5.3
COX5.4                .......... .......... .......... .......... .......... .......... .......... .......... G......... ..........  COX5.4
COX5.5                .......... .......... .......... .......... .......... .......... .......... .......... G......... ..........  COX5.5
COX5.6                .......... .......... .......... .......... .......... .......... .......... .......... G......... ..........  COX5.6
COX5.7                .......... .......... .......... .......... .......... .......... .......... .......... G......... .......T..  COX5.7
COX5.8                .......... .......... .......... .......... .......... .......... .......... .......... G......... ..........  COX5.8
COX5.Dugway_5J108-111 .......... .......... .......... .......... .......... .......... .......... .......... G......... ..........  COX5.Dugway_5J108-111

                             110        120        130        140        150        160        170        180        190        200
COX5.1                ACGTATTGTC CCTCTCCCCC TCGAGGAGAG GGTTAAGGAG AGGGGT---- --------TT TAAAGAACCT AACTGCTGCG GTTTTTTACC CTCTCCTAGC  COX5.1
COX5.2                ........C. .......... .......... .......... ....T.---- --------.. .......... .......... .......... ..........  COX5.2
COX5.3                .......... .......... .......... .......... ....T.AAGG AGAGGGGT.. .......... .......... .......... ..........  COX5.3
COX5.4                .......... .......... .......... ..-------- ---------- ------GT.. .......... .......... .......... ..........  COX5.4
COX5.5                .......... .......... .......... .......... ....T.---- --------.. .......... .......... .......... ..........  COX5.5
COX5.6                .......... .......... .......... .......... ......---- --------.. .......... .......... .......... ..........  COX5.6
COX5.7                .......... .......... .......... .......... ....T.---- --------.. .......... .......... .......... ..........  COX5.7
COX5.8                .......... .......... .......... .......... ....T.---- --------.. .......... .......... .......... ..........  COX5.8
COX5.Dugway_5J108-111 .......... .......... .......... .......... ......---- --------.. .......... .......... .......... ..........  COX5.Dugway_5J108-111

                             210        220        230        240        250        260        270        280        290        300
COX5.1                CTCTCCCGCA GGCGGGAGGG GAATAGCGGA AGGAGGCATT GAGGATTTAT ACAACTCCTT TT-AGTGCCA TCCAAGTTCT TTTTTCTGAA ACACTATCAA  COX5.1
COX5.2                .......... .......... .......... .......... .......... .......... ..T....... .......... .......... ..........  COX5.2
COX5.3                .......... .......... .......... .......... .......... .......... ..T....... .......... .......... ..........  COX5.3
COX5.4                .......... .......... .......... .......... .......... .......... ..T....... .......... .......... ..........  COX5.4
COX5.5                .......... .......... .......... .......... .......... .......... ..T......G .......... .......... ..........  COX5.5
COX5.6                .......... .......... .......... .......... .......... .......... ..T....... .......... .......... ..........  COX5.6
COX5.7                .......... .......... .......... .......... .......... .......... ..T....... .......... .......... ..........  COX5.7
COX5.8                .......... .......... .......... .......... .......... .......... ..T....... .......... .......... ..........  COX5.8
COX5.Dugway_5J108-111 .........C T......... .......... .......... .......... .......... ..T....... .......... .......... ..........  COX5.Dugway_5J108-111

                             310        320        330        340        350        360
COX5.1                GGTTGGGATC GATATGTTAA CAACACGATT AAAAATTATT CTTTCAATCA TCTCGCTTTC TTTGTT  COX5.1
COX5.2                .......G.. .......... .......... .......... T......... .......... ......  COX5.2
COX5.3                .......G.. .......... .......... .......... .......... .......... ......  COX5.3
COX5.4                .......G.. .......... .......... .......... .......... .......... ......  COX5.4
COX5.5                .......G.. .......... .......... .......... .......... .......... ......  COX5.5
COX5.6                .......G.. .......... .......... .......... .......... .......... ......  COX5.6
COX5.7                .......G.. .......... .......... .......... .......... .......... ......  COX5.7
COX5.8                .......G.. .......... .......... .......... .......... .......... ......  COX5.8
COX5.Dugway_5J108-111 .......G.. .......... .......... .......... .......... .......... ......  COX5.Dugway_5J108-111
```

```
                        10         20         30         40         50         60         70         80         90         100
                ....|....| ....|....| ....|....| ....|....| ....|....| ....|....| ....|....| ....|....| ....|....| ....|....|
COX18.1         GTGGCATTGA CGAAGTTTTG AAGAGGGGAG TGCAGTAGTT ATACCCCTAT CAAAAATTTA AAGCCCGTCG TTCCGCGCAG ACGCATAGGC GTCAACTTAA  COX18.1
COX18.2         .......... .......... .......... C..G...... .......... .......... .........A .......... .......... ..........  COX18.2
COX18.3         .......... .......... .......... C..G...... .......... .......... .........A .......... .......... ..........  COX18.3
COX18.4         .......... .......... .......... C......... .......... .......... .........A .......... .......... ..........  COX18.4
COX18.5         .......... .......... .......... .......... .......... .......... .........A .......... .......... ..........  COX18.5
COX18.6         .......... .......... .......... .......... .......... .......... .........A .......... .......... ..........  COX18.6
COX18.7         .......... .......... .......... .......... .......... .......... .......C.A .......... .......... ..........  COX18.7
COX18.8         .......... .......... .......... C..G...... .......... .......... .........A .......... .......... ..........  COX18.8
COX18.9         .......... .......... .......... C..G...... .......... .......... .........A .......... .......... ..........  COX18.9
COX18.10        .......... .......... .......... C..G...... .......... .......... .........A .......... .......... ..........  COX18.10
COX18.Dugway_5J108-111 .......... .......... .......... C......... .......... .......... .........A .....A.... .......... ..........  COX18.Dugway_5J108-111

                        110        120        130        140        150        160        170        180        190        200
                ....|....| ....|....| ....|....| ....|....| ....|....| ....|....| ....|....| ....|....| ....|....| ....|....|
COX18.1         AGAGAGCGAG GTAAAGAGGC AATATCAAGT AGTTGAAAAA ATATCCGTCG TTCCCGTGAC TTCGGGCGTG AAGGTTTTGT ATGCTTCAAT ACTACAGCAG  COX18.1
COX18.2         .......... .......... .......... .......... .......... .......... .....C.... .......... .......... ..........  COX18.2
COX18.3         .......... .......... ........-- ---------- ---------- ---....... .....C.... .......... .......... ..........  COX18.3
COX18.4         .......... .......... .......... .......... .......... .......... .....C.... .......... .......... ..........  COX18.4
COX18.5         .......... .......... .......... .......... .......... .......... .....C.... .......... .......... ..........  COX18.5
COX18.6         .......... .......... .......... .......... .......... .......... .....C.... .......... .......... ..........  COX18.6
COX18.7         .......... .......... .......... .......... .......... .......... .....C.... .......... .......... ..........  COX18.7
COX18.8         .......... .......... .......... .......... .......... .......... .....C.... .......... .......... ..........  COX18.8
COX18.9         .......... .......... .......... .......... .......... .......... .....C.... .......... .......... ..........  COX18.9
COX18.10        .......... .......... .......... .......... .......... .......... .....C.... .......... .......... ..........  COX18.10
COX18.Dugway_5J108-111 .......... .......... .......... .......... .......... .......... .....C.... .......... .......... .G........  COX18.Dugway_5J108-111

                        210        220        230        240        250        260        270        280        290        300
                ....|....| ....|....| ....|....| ....|....| ....|....| ....|....| ....|....| ....|....| ....|....| ....|....|
COX18.1         ACGGCCTCGC GGGAGGACGA CGGGTTTTAA GTTTTTCACG CTTCACCATG GGTGGGTAAT ATTAGGATCC GTGGTAAGCT TTGAAAGGAG CAAGGCGAAA  COX18.1
COX18.2         .......... .......... .......... .......... .......... .......... ........T. .......... .......... ..........  COX18.2
COX18.3         .......... .......... .......... .......... .......... .......... .......... .......... .......... ..........  COX18.3
COX18.4         .......... .......... .......... .......... .......... .......... .......... .......... .......... ..........  COX18.4
COX18.5         .......... .......... .......... .......... .......... .......... .......... .......... .......... ..........  COX18.5
COX18.6         .......... .......... .......... .......... .......... .......... .......... .......... .......... ..........  COX18.6
COX18.7         .......... .......... .......... .......... .......... .......... .......... .......... .......... ..........  COX18.7
COX18.8         .......... .......... .......... .......... .......... .......... .......... .......... .......... ..........  COX18.8
COX18.9         .......... .......... .......... .......... .......... .......... ........T. .......... .......... ..........  COX18.9
COX18.10        .......... .......... .......... .......... .C........ .......... .......... .......... .......... ..........  COX18.10
COX18.Dugway_5J108-111 .......... .......... .......... .......... .......... .......... .......... .......... .......... ..........  COX18.Dugway_5J108-111

                        310        320        330        340        350        360        370        380        390        400
                ....|....| ....|....| ....|....| ....|....| ....|....| ....|....| ....|....| ....|....| ....|....| ....|....|
COX18.1         GAGGGCATCA ACTTACAGTC ATTAGAGTTT TATGCTTAAG TTGGCGCTTC TGTGATTGTG TTGGAATAAC GAAGGCCAAA AAAGGGAGGC GCTGGGAGTT  COX18.1
COX18.2         .......... .......... .......... .......... .......... .......A.. .......... .......... .......... ..........  COX18.2
COX18.3         .......... .......... .......... .......... .......... .......... .......... .......... .......... ..........  COX18.3
COX18.4         ...A...... .......... .......... .......... .......... .......... .......... .......... .......... ..........  COX18.4
COX18.5         .......... .......... .......... .......... .......... .......... .......... .....T.... .......... ..........  COX18.5
COX18.6         .......... .......... .......... .......... .......... .......... .......... .......... .......... ..........  COX18.6
COX18.7         .......... .......... .......... .......... .......... .......... .......... .......... .......... ..........  COX18.7
COX18.8         .......... .......... .......... .......... .......... .......... .......... .......... .......... ..........  COX18.8
COX18.9         .......... .......... .......... .......... .......... .......A.. .......... .......... .......... ..........  COX18.9
COX18.10        .......... .......... .......... .......... .......... .......... .......... .......... .......... ..........  COX18.10
COX18.Dugway_5J108-111 .......... .......... .......... .......... .......... .......... .......... .......... .......... ..........  COX18.Dugway_5J108-111

                        410        420        430        440        450        460        470        480        490        500
                ....|....| ....|....| ....|....| ....|....| ....|....| ....|....| ....|....| ....|....| ....|....| ....|....|
COX18.1         TTTACTACCC TTACTTGATT TAAACCGAGA CATCTTTTTA AAGCACTATT AATTTTTGGG GAACTTAATG CGGCAAATCG TTTTAGATAC GGAAACCACC  COX18.1
COX18.2         .......... .......... .......... .......... ........C. .......... .......... .......... .......... ..........  COX18.2
COX18.3         .......... .......... .......... .......... .......... .......... .......... .......... .......... ..........  COX18.3
COX18.4         .......... .......... .......... .......... .......... .......... .......... .......... .......... ..........  COX18.4
COX18.5         .......... .......... .......... .......... .......... .......... .......... .......... .......... ..........  COX18.5
COX18.6         .......... .......... .......... .......... .......... .......... .......... .......... .......... ..........  COX18.6
COX18.7         .......... .......... .......... .......... .......... .......... .......... .......... .......... ..........  COX18.7
COX18.8         .......... .......... .......... .......... .......... .......... .......... .......... .......... ..........  COX18.8
COX18.9         .......... .......... .......... .......... .......... .......... .......... .......... .......... ..........  COX18.9
COX18.10        .......... .......... .......... .......... .......... .......... .......... .......... .......... ..........  COX18.10
COX18.Dugway_5J108-111 .......... .......... .......... .......... .......... .......... .......... .......... .......... ..........  COX18.Dugway_5J108-111

                        510
                ....|....| ....|.
COX18.1         GGCCTTGTGC CGGAAG  COX18.1
COX18.2         .......... ......  COX18.2
COX18.3         .......... ......  COX18.3
COX18.4         .......... ......  COX18.4
COX18.5         .......... ......  COX18.5
COX18.6         .......... ......  COX18.6
COX18.7         .......... ......  COX18.7
COX18.8         .......... ......  COX18.8
COX18.9         .......... ......  COX18.9
COX18.10        .......... ......  COX18.10
COX18.Dugway_5J108-111 .......... ......  COX18.Dugway_5J108-111
```

```
                              10         20         30         40         50         60         70         80         90        100
                     ....|....| ....|....| ....|....| ....|....| ....|....| ....|....| ....|....| ....|....| ....|....| ....|....|
COX20.1              AAATAATAGC AGCTATAGGC ATTAAAGTCC CGTTAAACAA ATGACCCGCT ATTGCGATAA CTGCCGAAGA TAGCGCCAAT CGAAATGGTG CCCAGTAGGG  COX20.1
COX20.2              .......... .......... .......... .......... .......... .......... .......... .......... .......... ..........  COX20.2
COX20.3              .......... .......... .......... .......... .......... .......... .......... .......... .......... ..........  COX20.3
COX20.4              .......... .......... .......... .......... .......... .......... .......... .......... .......... ..........  COX20.4
COX20.5              .......... .......... .......... .......... .......... .......... .......... .......... .......... ..........  COX20.5
COX20.6              .......... .......... .......... .......... .......... .......... .......... .......... .......... ..........  COX20.6
COX20.7              .......... .......... .......... .......... .......... .......... .......... .......... .......... ..........  COX20.7
COX20.8              .......... .......... .......... .......... .......... .......... .......... .......... .......... ..........  COX20.8
COX20.Dugway_5J108-111 .......... .......... .......... .......... .......... .......... .......... .......... .......... ..........  COX20.Dugway_5J108-111

                             110        120        130        140        150        160        170        180        190        200
                     ....|....| ....|....| ....|....| ....|....| ....|....| ....|....| ....|....| ....|....| ....|....| ....|....|
COX20.1              CAGAAGACGT TTCCCTGACT TCGGGATGGG TATTCAAGGC TTGTGAGATA AACGGCCCTA ATGCAATCGC TGAACCTGCC GCAAAGAGGG ACATTACCGC  COX20.1
COX20.2              .........C .C........ .......... .......... .......... .......... .......... .......... .......... ..........  COX20.2
COX20.3              .......... .C........ .......... .......... .......... .......... .......... .......... .......... ..........  COX20.3
COX20.4              .......... .C........ .......... .......... .......... .......... .......... .......... .......... ..........  COX20.4
COX20.5              .......... .C........ .......... .......... .......... .......... .......... .......... .......... ..........  COX20.5
COX20.6              .......... .C........ .......... .......... .......... .......... .......... .......... .......... ..........  COX20.6
COX20.7              .......... .C........ .......... .......... .......... .......... .......... .......... .......... ..........  COX20.7
COX20.8              .......... .C........ .......... .......... .......... .......... .......... .......... .......... ..........  COX20.8
COX20.Dugway_5J108-111 .......... .C........ .......... .......... .......... ....A..... .......... .......... .......... ..........  COX20.Dugway_5J108-111

                             210        220        230        240        250        260        270        280        290        300
                     ....|....| ....|....| ....|....| ....|....| ....|....| ....|....| ....|....| ....|....| ....|....| ....|....|
COX20.1              CGTAATTAAA TTGGGGCTGT TAGGTAATAT GAAGTGGGAT GACAACAGTA ATACGGCGGC TAGCGATACG ATAAATAATC CAATACGTTG GGAGGCTTGT  COX20.1
COX20.2              .......... .......... .......... .......... .......... .......... .......... .......... .......... ..........  COX20.2
COX20.3              .......... .......... .......... .......... .......... .......... .......... .......... .....T.... ..........  COX20.3
COX20.4              .......... .......... .......... .......... .......... .......... .......... .......... .......... ..........  COX20.4
COX20.5              .......... .......... .......... .......... .......... .......... .......... .......... .......... ..........  COX20.5
COX20.6              .......... .......... .......... .......... .......... .......... .......... .......... .......... ..........  COX20.6
COX20.7              .......... .......... .......... .......... .......... .......... ...T...... .......... .......... ..........  COX20.7
COX20.8              .......... .....A.... .......... .......... .......... .......... .......... .......... .......... ..........  COX20.8
COX20.Dugway_5J108-111 .......... .......... .......... .......... .......... .......... .......... .......... .......... ..........  COX20.Dugway_5J108-111

                             310        320        330        340        350        360        370        380        390        400
                     ....|....| ....|....| ....|....| ....|....| ....|....| ....|....| ....|....| ....|....| ....|....| ....|....|
COX20.1              ATAGAAAATC GCTTCAGCAT ATAAGCAGCG ATAAAGCTAA ACGCCAAAAA CGTCAACATA ATGATTGCTT CATAATAACC TACCTGATGC TGGGTTACTC  COX20.1
COX20.2              .......... .......... .......... .......... .......... .......... .......... .......... .......... ..........  COX20.2
COX20.3              .......... .......... .......... .......... .......... .......... .......... .......... .......... ..........  COX20.3
COX20.4              .......... .......... .......... .......... .......... .......... .......... .......... .......... ..........  COX20.4
COX20.5              .......... .......... .......... .......... .......... .......... .......... .......... .......... ..........  COX20.5
COX20.6              .......... .......... .......... .......... .......... .......... .......... .......... .......... ..........  COX20.6
COX20.7              .......... .......... .......... .......... .......... .......... .......... .......... .......... ..........  COX20.7
COX20.8              .......... .......... .......... .......... .......... .......... .......... .......... .......... ..........  COX20.8
COX20.Dugway_5J108-111 .......... .......... .......... .......... .......... .......... .......... .......... .......... ..........  COX20.Dugway_5J108-111

                             410        420        430        440        450        460        470        480        490        500
                     ....|....| ....|....| ....|....| ....|....| ....|....| ....|....| ....|....| ....|....| ....|....| ....|....|
COX20.1              CAAGATGGTT AACAAAGAGT AAGGATAAAT TAGCAATATA TACAATGATG CCTGCGTACA TCAAACAAGG AATCGTACCT AAAA--TAAT GAATTTTTTA  COX20.1
COX20.2              .......... .......... .......... .......... .......... .......... .......... .......... ....AA.... ..........  COX20.2
COX20.3              .......... .......... .......... .......... .......... .......... .......... .......... ....--.... ..........  COX20.3
COX20.4              .......... .......... .......... .......... .......... .......... .......... .......... ....AA.... ..........  COX20.4
COX20.5              .......... .......... .......... .......... .......... .......... .......... .......... ....AA.... ..........  COX20.5
COX20.6              .......... .......... .......... .......... .......... .......... .......... .......... ....--.... ..........  COX20.6
COX20.7              .......... .......... .......... .......... .......... .......... .......... .......... ....--.... ..........  COX20.7
COX20.8              .......... .......... .......... .......... .......... .......... .......... .......... ....--.... ..........  COX20.8
COX20.Dugway_5J108-111 .......... .......... .......... .......... .......... .......... .......... .......... ...---.... ..........  COX20.Dugway_5J108-111

                             510        520        530        540        550        560        570        580
                     ....|....| ....|....| ....|....| ....|....| ....|....| ....|....| ....|....| ....|....| ....|...
COX20.1              TCTCGTAGTA AATAGAAATA GGATTGAATC ATGTTTTTTA ACTTTACTGC CCCCC-TGCA TGTTTATATT TTTTAGGCTC TTTAAAAA  COX20.1
COX20.2              .......... .......... .......... .......... .......... .....-.... .......... .......... ........  COX20.2
COX20.3              .......... .......... .......... .......... .......... .....-.... .......... .......... ........  COX20.3
COX20.4              .......... .......... .......... .......... .......... .....C.... .......... .......... ........  COX20.4
COX20.5              .......... .......... .......... .......... .......... .....-.... .......... .......... ........  COX20.5
COX20.6              .......... .......... .......... .......... .......... .....-.... .......... .......... ........  COX20.6
COX20.7              .......... .......... .......... .......... .......... .....-.... .......... .......... ........  COX20.7
COX20.8              .......... .......... .......... .......... .......... .....-.... .......... .......... ........  COX20.8
COX20.Dugway_5J108-111 .......... .......... .......... .......... .......... ....--.... .......... .......... ........  COX20.Dugway_5J108-111
```

```
                              10         20         30         40         50         60         70         80         90        100
                      ....|....| ....|....| ....|....| ....|....| ....|....| ....|....| ....|....| ....|....| ....|....| ....|....|
COX22.1               TGAGGTTTAG AATCAACGAC AGTGGGTGAA TAGATTACGC CTTCCATTAT TGATTTATTG CCAAGAGGGG TGCTGTCGGG TGGTGCTCCC CTGTAGTGCG  COX22.1
COX22.2               .......... .......... .......... .......... .......... .......... .......... .......... .......... ..........  COX22.2
COX22.3               .......... .......... .......... .......... .......... .......... .......... .......... .......... T.........  COX22.3
COX22.4               .......... .......... .......... .......... .......... .......... .......... .......... .......... T.........  COX22.4
COX22.5               .......... .......... .......... .......... .......... .......... .......... .......... .......... T.........  COX22.5
COX22.6               .......... .......... .......... .......... .......... .......... .......... .......... .......... ..........  COX22.6
COX22.7               .......... .......... .......... .......... .......... .......... .......... .......... .......... ..........  COX22.7
COX22.8               .......... .......... .......... .......... .......... .......... .......... .......... .......... ..........  COX22.8
COX22.Dugway_5J108-111 .......... .......... .......... .......... .......... .......... .......... .......... .......... ..........  COX22.Dugway_5J108-111

                             110        120        130        140        150        160        170        180        190        200
                      ....|....| ....|....| ....|....| ....|....| ....|....| ....|....| ....|....| ....|....| ....|....| ....|....|
COX22.1               GAGAAAATAT TGAACGAAAA TTACATAAGG CGTTAAACCA CTGTATTCGT TGTTGTTGAT TCATAATCGT ATTCCTCAGC TATCACTCAC TTTTTGCTTA  COX22.1
COX22.2               .......... .......C.. .......... .......TG. .......... .......... .......... .......... .......... ..........  COX22.2
COX22.3               .......... .......C.. .......... .......T.. .......... .......... ........A. .......... .......... ..........  COX22.3
COX22.4               .......... .......C.. .......... .......T.. .......... .......... .T........ .......... .......... ..........  COX22.4
COX22.5               .......... .......C.. .......... .......T.. .......... .......... .......... .......... .......... ..........  COX22.5
COX22.6               .......... .......C.. .......... .......... .......... .......... .......... .......... .......... ..........  COX22.6
COX22.7               .......... .......... .......... .......... .......... .......... .......... .......... .......... ..........  COX22.7
COX22.8               .......... .......... .......... .......... .......... .......... .......... .......... .......... ..........  COX22.8
COX22.Dugway_5J108-111 .......... .......C.. .......... .......... .......... .......... .......... .......... .......... ..........  COX22.Dugway_5J108-111

                             210        220        230        240        250        260        270        280        290        300
                      ....|....| ....|....| ....|....| ....|....| ....|....| ....|....| ....|....| ....|....| ....|....| ....|....|
COX22.1               GCGCTAACCA TTAACAATGT CTATATTTTA CACAGATCCA GGTTCGGTTT GAAGGACTGG TTTGTCGTAT TTCGCTCGCG TATGGGCTGC CATTTATGCG  COX22.1
COX22.2               .......T.. .......... .......... .......... .......... .......... .......... .......... .......... ..........  COX22.2
COX22.3               .......... .......... .......... .......... .......... .......... .......... .......... .......... ..........  COX22.3
COX22.4               .......... .......... .......... .......... .......... .......... .......... .......... .......... ..........  COX22.4
COX22.5               .......... .......... .......... .......... .......... .......... .......... .......... .......... ..........  COX22.5
COX22.6               .......... .......... .......... .......... .......... .......... .......... .......... .......... ..........  COX22.6
COX22.7               .......... .......... .......... .......... .......... .......... .......... .......... .......... .........A  COX22.7
COX22.8               .......... .......... .......... .......... .......... .......... .......... .......... .......... ..........  COX22.8
COX22.Dugway_5J108-111 .......... .......... .......... .......... .......... .......... .......... .......... .......... ..........  COX22.Dugway_5J108-111

                             310        320        330        340
                      ....|....| ....|....| ....|....| ....|....| .
COX22.1               CTCGCT-CTG AATCGACAGA GTCTAGTGTA TTGGCTTGAT G  COX22.1
COX22.2               ......T... .......... .......... .......... .  COX22.2
COX22.3               ......T... .......... .......... .......... .  COX22.3
COX22.4               ......T... .......... .......... .......... .  COX22.4
COX22.5               ......T... .......... .......... .......... .  COX22.5
COX22.6               ......T... .......... .......... .......... .  COX22.6
COX22.7               ......T... .......... .......... .......... .  COX22.7
COX22.8               ......T... .......... .......... .......... .  COX22.8
COX22.Dugway_5J108-111 ......T... .......... .......... .......... .  COX22.Dugway_5J108-111
```

```
                                 10         20         30         40         50         60         70         80         90        100
                        ....|....| ....|....| ....|....| ....|....| ....|....| ....|....| ....|....| ....|....| ....|....| ....|....|
COX37.1                 AAACTTTACA GCTTGGTAGC CTTGGATTTC CTCTATTTGG GAATCGGAAT TAACTTTCTG TTCATTACTT CGCATATTAT TTTTCTCCAA TTGAAGTTAC  COX37.1
COX37.2                 .......... .......... .......... .......... ....A..... .......... .......... .......... .......... .........T  COX37.2
COX37.3                 .......... .......... .......... .......... .......... .......... .......... .......... .......... .........T  COX37.3
COX37.4                 .......... .......... .......... .......... .......... .......... .......... .......... .......... .........T  COX37.4
COX37.5                 .......... .......... .......... .......... ....A..... .......... .......... .......... .......... .........T  COX37.5
COX37.6                 .......... .......... .......... ..G....... ....A..... .......... .......... .......... .......... .........T  COX37.6
COX37.7                 .......... .......... .......... ..A....... ....A..... .......... .......... .......... .......... .........T  COX37.7
COX37.8                 .......... .......... .......... .......... .......... .......... .......... .......... .......... .........T  COX37.8
COX37.9                 .......... .......... .......... .......... .......... .......... .......... .......... .......... .........T  COX37.9
COX37.Dugway_5J108-111  .......... .......... .......... .......... .......... .......... .......... .......... .......... .........T  COX37.Dugway_5J108-111

                                110        120        130        140        150        160        170        180        190        200
                        ....|....| ....|....| ....|....| ....|....| ....|....| ....|....| ....|....| ....|....| ....|....| ....|....|
COX37.1                 CGCCACGGGG TTATCAGTTC CAAGGGCTCA CTCAGGTATA ATGTCACTAG TGTATTATCA GGAAATTTCT GGTTAAACCT TTCAAGGAGG TAAAATGAAG  COX37.1
COX37.2                 .......... .......... ......T... .......... .......... .......... .......... .......... .......... ..........  COX37.2
COX37.3                 .......... .......... .......... .......... .......... .......... .......... .......... .......... ..........  COX37.3
COX37.4                 .......... .......... .......... .......... .......... .......... .......... .......... .......... ..........  COX37.4
COX37.5                 .......... .......... .......... .......... .......... .......... .......... .......... .......... ..........  COX37.5
COX37.6                 .......... .......... .......... .......... .......... .......... .......... .......... .......... ..........  COX37.6
COX37.7                 .......... .......... .......... .......... .......... .......... .......... .......... .......... ..........  COX37.7
COX37.8                 .......... .......... .......... .......... .......... .......... .......... .......... .......... ..........  COX37.8
COX37.9                 .......... .......... .......... .......... .......... .......... .......... .......... .......... C.........  COX37.9
COX37.Dugway_5J108-111  .......... .......... .......... .......... .......... .......... .......... .......... .......... ..........  COX37.Dugway_5J108-111

                                210        220        230        240        250        260        270        280        290        300
                        ....|....| ....|....| ....|....| ....|....| ....|....| ....|....| ....|....| ....|....| ....|....| ....|....|
COX37.1                 CTTTAGATTA AAAGCTCCCA GATAAACTTA AAGAGGGGTT AAGTCGAGTA TATAAGTTGA ATCCGAATTA ATTTTTCACC CAGCATCATC GCTTACAATG  COX37.1
COX37.2                 .......... .......... .......... .......... .......... .......... .......... .......... .......... ..........  COX37.2
COX37.3                 .......... .......... .......... ........C. .......... .......... .......... .......... .......... ..........  COX37.3
COX37.4                 .......... .......... .......... .......... .......... .......... .......... .......... .......... ..........  COX37.4
COX37.5                 .......... .......... .......... .......... .......... .......... .......... .......... .......... ..........  COX37.5
COX37.6                 .......... .......... .......... .......... .......... .......... .......... .......... .......... ..........  COX37.6
COX37.7                 .......... .......... .......... .......... .......... .......... .......... .......... .......... ..........  COX37.7
COX37.8                 .......... ....T..... .......... .......... .......... .......... .......... .......... .......... ..........  COX37.8
COX37.9                 .......... .......... .......... .......... .......... .......... .......... .......... .......... ..........  COX37.9
COX37.Dugway_5J108-111  .......... .......... .......... .......... .......... .......... .......... .......... .......... ..........  COX37.Dugway_5J108-111

                                310        320        330        340        350        360        370        380        390        400
                        ....|....| ....|....| ....|....| ....|....| ....|....| ....|....| ....|....| ....|....| ....|....| ....|....|
COX37.1                 GCGAGGTCAA CATTAAAACC ATGCGACAAA ATACTTTCAA CCAAGGCTTC TTCCGTCGCA ATTCTGCAAT GATGAGAACT AATAATATCC AGAGTCTTTA  COX37.1
COX37.2                 .......... .......... .......... .......... .......... .--....... .......... .......... .......... ..........  COX37.2
COX37.3                 .......... .......... .......... .......... .......... .......... .......... .......... .......... ..........  COX37.3
COX37.4                 .......... .......... .......... .......... .......... .......... .......... .......... .......... ..........  COX37.4
COX37.5                 .......... .......... .......... .......... .......... .--....... .......... .......... .......... ..........  COX37.5
COX37.6                 .......... .......... .......... .......... .......... .--....... .......... .......... .......... ..........  COX37.6
COX37.7                 .......... .......... .......... .......... .......... .--....... .......... .......... .......... ..........  COX37.7
COX37.8                 .......... .......... .......... .......... .......... .......... .......... .......... .......... ..........  COX37.8
COX37.9                 .......... .......... .......... .......... .......... .......... .......... .......... .......... ..........  COX37.9
COX37.Dugway_5J108-111  .......... .......... .......... .......... .......... .......... .......... .......... .......... ..........  COX37.Dugway_5J108-111

                                410        420
                        ....|....| ....|....| ..
COX37.1                 AATGGGGTTG CCAAGCTTCT TT  COX37.1
COX37.2                 .......... .......... ..  COX37.2
COX37.3                 .......... .......... ..  COX37.3
COX37.4                 .......... .......... ..  COX37.4
COX37.5                 .......... .......... ..  COX37.5
COX37.6                 .......... .......... ..  COX37.6
COX37.7                 .......... .......... ..  COX37.7
COX37.8                 .......... .......... ..  COX37.8
COX37.9                 .......... .......... ..  COX37.9
COX37.Dugway_5J108-111  .......... .......... ..  COX37.Dugway_5J108-111
```

```
                                  10         20         30         40         50         60         70         80         90        100
COX51.1                  TTCAAAAAAA TTATTCGAAA AATACAGCAG CTCTCAAGTT GAGAGAATAG TGGGTTTTAC TAATTTTTCA TAATATTCGT ACGATAATTT AAGTAATTGT  COX51.1
COX51.2                  .......... .......... .......... .......... .......... .......... .........G ....C..... .......... ..........  COX51.2
COX51.3                  .......... .......... .......... .......... .......... .......... ......C..G ....C..... .......... ..........  COX51.3
COX51.4                  .......... .......... .......... .......... .......... .......... .......... ....C..... .......... ..........  COX51.4
COX51.5                  .......... .......... .......... .......... .......... .......... .......... ....C..... .......... ..........  COX51.5
COX51.6                  .......... .......... .......... .......... .......... .......... .......... ....C..... .......... ..........  COX51.6
COX51.7                  .......... .......... .......... .......... .......... .......... .......... ....C..... .......... ..........  COX51.7
COX51.8                  .......... .......... .......... .......... .......... .......... .......... ....C..... .......... ..........  COX51.8
COX51.9                  .......... .......... .......... .......... .......... .......... .......... ....C..... .......... ..........  COX51.9
COX51.10                 .......... .......... .......... .......... .......... .......... .......... ....C..... .......... ..........  COX51.10
COX51.11                 .......... .......... .......... .......... .......... .......... .......... ....C..... .......... ..........  COX51.11
COX51.Dugway_5J108-111   .......... .......... .......... .......... .......... .......... .......... ....C..... .......... ..........  COX51.Dugway_5J108-111

                                 110        120        130        140        150        160        170        180        190        200
COX51.1                  GAGTGAGTGC CGGCATTAAA AACAATTTCT TTTTCTTCGC TCAACTCTTT GGAAAGGTAA ACTTTCATAT CGAATAAATT GCCAAA-GGT GGCATTGCCC  COX51.1
COX51.2                  .......... .......... .......... .......... .......... .......... .......... .......... ......A... ..........  COX51.2
COX51.3                  .......... .......... .......... .......... .......... .......... .......... .......... ......A... ..........  COX51.3
COX51.4                  .....----. .......... .......... .......... .......... .......... .......... .......... ......A... ..........  COX51.4
COX51.5                  .......... .......... .......... .......... .......... .......... .......... .......... ......A... ..........  COX51.5
COX51.6                  .......... .......... .......... .......... .......... .......... .......... .......... ......A... ..........  COX51.6
COX51.7                  .......... .......... .......... .......... .......... .......... .......... .......... ......A... ..........  COX51.7
COX51.8                  .......... .......... .......... .......... .......... .......... .......... .......... ......A... ..........  COX51.8
COX51.9                  .......... .......... .......... .......... .......... .......... .......... .......... ......A... ..........  COX51.9
COX51.10                 .......... .......... .......... .......... .......... .......... .......... .......... ......A... ..........  COX51.10
COX51.11                 .......... .......... .......... .......... .......... .......... .......... .......... ......A... ..........  COX51.11
COX51.Dugway_5J108-111   .......... .......... .......... .......... .......... .......... .......... .......... ......A... ..........  COX51.Dugway_5J108-111

                                 210        220        230        240        250        260        270        280        290        300
COX51.1                  CTAATTCACA GCCGGGAAAT CGTTCTTGAA ATTCGTATTC GCTGGCTAAT TCCGCTTTAT GGGCTCCAGT TTTTTGTTTT AATTTATCAA AGTCGACTTT  COX51.1
COX51.2                  .......... .......... .......... .......... .......... .......... .......... .......... .......... ..........  COX51.2
COX51.3                  .......... .......... .......... .......... .......... .......... .......... .......... .......... ..........  COX51.3
COX51.4                  .......... .......... .......... .......... .......... .......... .......... .....-...- .......... ..........  COX51.4
COX51.5                  .......... .......... .......... .......... .......... .......... .......... .....-..-- .......... ..........  COX51.5
COX51.6                  .......... .......... .......... .......... .......... .......... .......... .....-...- .......... ..........  COX51.6
COX51.7                  .......... .......... .......... .......... .......... .......... .......... .....-...- .......... ..........  COX51.7
COX51.8                  .......... .......... .......... .......... .......... .......... .......... .....-...- .......... ..........  COX51.8
COX51.9                  .......... .......... .......... .......... .......... .......... .......... .....-..-- .......... ..........  COX51.9
COX51.10                 .......... .......... .......... .......... .......... .......... .......... .....-...- .......... ..........  COX51.10
COX51.11                 .......... .......... .......... .......... .......... .......... .......... .....-.... .......... ..........  COX51.11
COX51.Dugway_5J108-111   ..G.....T. .......... .......... .......... .......... .......... .......... .......... .......... ..........  COX51.Dugway_5J108-111

                                 310        320        330        340        350        360        370        380        390        400
COX51.1                  ATCATCGCCC GGTAGCACCA CCATTGCGAG CTTATCATCG ATTTTAGCAA TAACACATTT CGCTAGCTTT TTAGCAGATA CGTGTGAAAT TTCCGCAATT  COX51.1
COX51.2                  .......... .......... .......... .......... .......... .......... .......... .......... .......... ..........  COX51.2
COX51.3                  .......... .......... .......... .......... .......... .......... .......... .......... .......... ..........  COX51.3
COX51.4                  .......... .......... .......... .......... .......... .......... .......... .......... .......... ..........  COX51.4
COX51.5                  .......... .......... .......... .......... .......... .......... .......... .......... .......... ..........  COX51.5
COX51.6                  .......... .......... .......... .......... .......... .....A.... .......... .......... .......... ..........  COX51.6
COX51.7                  .......... .......... .......... .......... .......... .......... .......... .......... .......... ..........  COX51.7
COX51.8                  .......... .......... .......... .......... .......... .......... .......... .......... .......... ..........  COX51.8
COX51.9                  .......... .......... .......... .......... .......... .......... .......... .......... .......... ..........  COX51.9
COX51.10                 .......... .......... .......... .......... .......... .......... .......... .......... .......... ..........  COX51.10
COX51.11                 .......... .......... .......... .......... .......... .......... .......... .......... .......... ..........  COX51.11
COX51.Dugway_5J108-111   .......... .......... .......... .......... .......... .......... .......... .......... .......... ..........  COX51.Dugway_5J108-111

                                 410        420        430        440        450        460        470        480        490        500
COX51.1                  TCTTGGGCAG TATAAGCTGG AGAATGATTA ATAGTGACAT ATTTCACTTT GTTTTTATCG AGAAATTCTT TCAATTTTTC AAGCGGCATA CGAAACCTCC  COX51.1
COX51.2                  .......... .......... .......... .......... .......... .......... .......... .......... .......... ..........  COX51.2
COX51.3                  .......... .......... .......... .......... .......... .......... .......... .......... .......... ..........  COX51.3
COX51.4                  .......... .......... .......... .......... .......... .......... .......... .......... .......... ..........  COX51.4
COX51.5                  .......... .......... .......... .......... .......... .......... .......... .......... .......... .A........  COX51.5
COX51.6                  .......... .......... .......... .......... .......... .......... .......... .......... .......... ..........  COX51.6
COX51.7                  .......... .......... .......... .......... .......... .......... .......... .......... .......... ..........  COX51.7
COX51.8                  .......... .......... .......... .......... .......... .......... .......... .......... .......... ..........  COX51.8
COX51.9                  ...C...... .......... .......... .......... .......... .......... .......... .......... .......... .A........  COX51.9
COX51.10                 .......... .......... .......... .......... .......... .......... .......... .......... .......... ..........  COX51.10
COX51.11                 .......... .......... .......... .......... .......... .......... .......... .......... .......... ..........  COX51.11
COX51.Dugway_5J108-111   .......... .......... .......... .......... .......... .......... .......... .......... .......... ..........  COX51.Dugway_5J108-111

                                 510        520        530        540        550        560        570        580        590        600
COX51.1                  CGACTTATCC CGTCTCTATA TAGAGAATTA TAGCTCATTA GGAAAATAGC AATAACCTTG AAAAGCCCAT CAGGCGTGCC TTTTCAAGGC CCCATTGACG  COX51.1
COX51.2                  .......... .......... .......... .......... .......... .......... .......... .......... .......... ..........  COX51.2
COX51.3                  .......... .......... .......... .......... .......... .......... .......... .......... .......... ..........  COX51.3
COX51.4                  .......... .......... .......... .......... .......... .......... .......... .......... .......... ..........  COX51.4
COX51.5                  .......... .......... .......... .......... .......... .......... .......... .......... .......... ..........  COX51.5
COX51.6                  .......... .......... .......... .......... .......... .......... .......... .......... .......... ..........  COX51.6
COX51.7                  .......... .......... .......... .......... .......... .......... .......... .......... .........T ..........  COX51.7
COX51.8                  .......... .......... .......... .......... .......... .......... .......... .......... .......... ..........  COX51.8
COX51.9                  .......... .......... .......... .......... .......... .......... .......... .......... .......... ..........  COX51.9
COX51.10                 .......... .......... .......... .......... .......... .......... .......... .......... .......... ..........  COX51.10
COX51.11                 .......... .......... .......... .......... .......... .......... .......... .......... .......... ..........  COX51.11
COX51.Dugway_5J108-111   .......... .......... .......... .......... .......... .......... .......... .......... .......... ..........  COX51.Dugway_5J108-111

                                 610        620        630
COX51.1                  CCTAACAGCT CCCGCATTTT GGCTTGAATC ATTTC  COX51.1
COX51.2                  .......... .......... .......... .....  COX51.2
COX51.3                  .......... .......... .......... .....  COX51.3
COX51.4                  .......... .......... .......... .....  COX51.4
COX51.5                  .......... .......... .......... .....  COX51.5
COX51.6                  .......... .......... .......... .....  COX51.6
COX51.7                  .......... .......... .......... .....  COX51.7
COX51.8                  .......... .......... .......... .....  COX51.8
COX51.9                  .......... .......... .......... .....  COX51.9
COX51.10                 .......... .......... .......... .....  COX51.10
COX51.11                 .......... .......... .......... .....  COX51.11
COX51.Dugway_5J108-111   .......... .......... .......... .....  COX51.Dugway_5J108-111
```

```
                              10         20         30         40         50         60         70         80         90        100
                     ....|....| ....|....| ....|....| ....|....| ....|....| ....|....| ....|....| ....|....| ....|....| ....|....|
COX56.1              CCCACAGCTA CTGTTGCAAT CAGAGCTAAG ACTATGTTTT TCATTATAAT TTAACTCTCA ACCTCTTGAT CTACTTTAAC TTTCAAAACT CTAACTATTC  COX56.1
COX56.2              .......... .......... .......... .......... .......... .......... .......... .......... .......... ..........  COX56.2
COX56.3              .......... .......... .......... .......... .......... .......... .......... .......... .......... ..........  COX56.3
COX56.4              .......... .......... .......... .....---.. .......... .......... .......... ...T...... .......... ......G...  COX56.4
COX56.5              .......... .......... .......... .....---.. .......... .......... .......... ...T...... .......... ......G...  COX56.5
COX56.6              .......... .......... .......... .....---.. .......... .......... .......... ...T...... .......... ......G...  COX56.6
COX56.7              .......... .......... .......... .....---.. .......... .......... .......... .......... .......... ..........  COX56.7
COX56.8              .......... .......... .......... .....---.. .......... .......... .......... .......... .......... ..........  COX56.8
COX56.9              .......... .......... .......... .....---.. .......... .......... .......... .......... .......... ..........  COX56.9
COX56.10             .........G .......... .......... .....---.. .......... .......... .......... .......... .......... ..........  COX56.10
COX56.11             .......... .......... .......... .......... .......... .......... .......... .......... .......... ..........  COX56.11
COX56.12             .......... .......... .......... .....---.. .......... .......... .......... ...T...... .......... ......G...  COX56.12
COX56.13             .......... .......... .......... .......... .......... .......... ..-....... .......... .......... ..........  COX56.13
COX56.Dugway_5J108-111 .......... .......... .......... .......... .......... .......... .......... .......... .......... ..........  COX56.Dugway_5J108-111

                             110        120        130        140        150        160        170        180        190        200
                     ....|....| ....|....| ....|....| ....|....| ....|....| ....|....| ....|....| ....|....| ....|....| ....|....|
COX56.1              CATAATCAGA GCCGGGAGTC AAGCTTCAGC CCTGCAGCGA GGAGCCATCT T-AGCTTAAG CGCTGGCGCG ATTCAAATCT CCTACTCAGA ATAATTCTTA  COX56.1
COX56.2              .......... .......... .......... .......... .......... .-........ .......... .......... .......... ..........  COX56.2
COX56.3              .......... .......... .......... .......... .......... .-........ .......... .......... .......... ..........  COX56.3
COX56.4              .......... .......... .......... .......... .......... .-........ .......... .......... .......... ..........  COX56.4
COX56.5              .......... .......... .......... .......... .......... .T........ .......... .......... .......... ..........  COX56.5
COX56.6              .......... .......... .......... .......... .......... .-........ .......... .......... .......... ..........  COX56.6
COX56.7              .......... .......... .......... .......... .......... .-........ .......... .......... .......... ..........  COX56.7
COX56.8              .......... .......... .......... .......... .......... .-........ .......... .......... .......... ..........  COX56.8
COX56.9              .......... .......... .......... .......... .......... .-........ .......... .......... .......... ..........  COX56.9
COX56.10             .......... .......... .......... .......... .......... .-........ .......... .......... .......... ..........  COX56.10
COX56.11             .......... .......... .......... .......... .......... .-........ ......T... .......... .......... ..........  COX56.11
COX56.12             .......... .......... .......... .......... .......... .-........ .......... .......... .......... ..........  COX56.12
COX56.13             .......... .......... .......... .......... .......... .-........ .....-T... .......... .......... ..........  COX56.13
COX56.Dugway_5J108-111 .......... .......... .......... .......... .......... .-........ .......... .......... .......... ..........  COX56.Dugway_5J108-111

                             210        220        230        240        250        260        270        280        290        300
                     ....|....| ....|....| ....|....| ....|....| ....|....| ....|....| ....|....| ....|....| ....|....| ....|....|
COX56.1              CTCTAAATTG CGCCTTAAAA TCTTAAGTAA GGATGAATTT AAGAAGAAAA ATACGGGGTT AAACTTTAGG CTTGCTCTAT CGCTAATCAG TATGATATAT  COX56.1
COX56.2              .......... .......... .......... .......... .......... .......... .......... .......... .......... ..........  COX56.2
COX56.3              .......... .......... .......... .......... .......... .......... .......... .......... .......... ..........  COX56.3
COX56.4              .......... .......... .......... .......... .......... .......... .......... .......... .......... ..........  COX56.4
COX56.5              .......... .......... .......... .......... .......... .......... .......... .......... .......... ..........  COX56.5
COX56.6              .......... .......... .......... .......... .......... .......... .......... .......... .......... ..........  COX56.6
COX56.7              .......... .......... .......... .......... .......... .......... .......... .......... .......... ..........  COX56.7
COX56.8              .......... .......... .......... .......... .......... .......... .......... .......... .......... ..........  COX56.8
COX56.9              .......... .......... .......... .......... .......... .......... .......... .......... .......... ..........  COX56.9
COX56.10             .......... .......... .......... .......... .......... .......... .......... .......... .......... ..........  COX56.10
COX56.11             .......... .......... ........C. .......... .......... .......... ...T...... .......... .......... ..........  COX56.11
COX56.12             .......... .......... .......... .......... .......... .......... .......... .......... .......... ..........  COX56.12
COX56.13             .......... .......... .......... .......... .......... .......... .......... .......... .......... ..........  COX56.13
COX56.Dugway_5J108-111 .......... .......... .......... .......... .......... .......... .......... .......... .......... ..........  COX56.Dugway_5J108-111

                             310        320        330        340        350        360        370        380        390        400
                     ....|....| ....|....| ....|....| ....|....| ....|....| ....|....| ....|....| ....|....| ....|....| ....|....|
COX56.1              ACCTTATCTA GTA-TAGTAG TGCTAGTAGT GCACAATTTA GCCATGATGG GACACAAAAA AATATGAGGT GACTTGTGAC AGGGTTGTTA CTCCACCCGA  COX56.1
COX56.2              .......... ...G...--- .......... .......... .......... .......... .......... .......... .......... ..........  COX56.2
COX56.3              .......... ...G...... .......... .......... .......... .......... .......... .......... .......... ..........  COX56.3
COX56.4              .....C.TA. T--------- ---....... .......... ....C..... .......... .......... .......... .......... ..........  COX56.4
COX56.5              .....C.TA. T--------- ---....... .......... ....C..... .......... .......... .......... .......... ..........  COX56.5
COX56.6              .....C.TA. T--------- ---....... .......... ....C..... .......... .......... .......... .......... ..........  COX56.6
COX56.7              .....C.TA. T--------- ---....... .......... .......... .......... .......... .......... .......... ..........  COX56.7
COX56.8              .....C.TA. T--------- ---....... A......... .......... A......... .......... .......... .......... ..........  COX56.8
COX56.9              .....C.TA. T--------- ---....... .......... ....C..... .......... .......... .......... .......... ..........  COX56.9
COX56.10             .....C.--- ---------- ----...... .......... .......... .......... .......... .......... .......... ..........  COX56.10
COX56.11             .......T.. ..--...... ..-....... .......... .......... .......... .......... .......... .......... ..........  COX56.11
COX56.12             .....CCTA. T.-------- ----...... .......... ....C..... .......... .......... .......... .......... ..........  COX56.12
COX56.13             .......... ...G...--- .......... .......... .......... .......... .......... .......... .......... ..........  COX56.13
COX56.Dugway_5J108-111 .......T.. ..-------- ----...... .......... .......... .......... .......... .......... .......... ..........  COX56.Dugway_5J108-111

                             410        420        430        440        450
                     ....|....| ....|....| ....|....| ....|....| ....|....| ....
COX56.1              AATTCTAAGA GGAGAGACAA CCATGACGTC CAAGCTGGTC ATTTCCGCGT TGGG  COX56.1
COX56.2              .......... .......... .......... .......... .......... ....  COX56.2
COX56.3              .......... .......... .......... .......... .......... ....  COX56.3
COX56.4              .......... .......... .......... .......... .......... ....  COX56.4
COX56.5              .......... .......... .......... .......... .......... ....  COX56.5
COX56.6              .......... .......... .......... .......... .......... ....  COX56.6
COX56.7              .......... .......... .......... .......... .......... ....  COX56.7
COX56.8              .......... .......... .......... .......... .......... ....  COX56.8
COX56.9              .......... .......... .......... .......... .......... ....  COX56.9
COX56.10             .......... .......... .......... .......... .......... ....  COX56.10
COX56.11             .......... .......... .......... .......... .......... ....  COX56.11
COX56.12             .......... .......... .......... .......... .......... ....  COX56.12
COX56.13             .......... .......... .......... .......... .......... ....  COX56.13
COX56.Dugway_5J108-111 .......... .......... .......... .......... .......... ....  COX56.Dugway_5J108-111
```

```
                                 10         20         30         40         50         60         70         80         90        100
                         ....|....| ....|....| ....|....| ....|....| ....|....| ....|....| ....|....| ....|....| ....|....| ....|....|
COX57.1                  TGGAAATGGA AGGCGGATTC AAAACCTGGA CCGAGCACAA ACTTGAAACC GAGAAATCAT ATTGACTTAG CCCCCAACCT CAAGCACTAT AAGCCCTCTG   COX57.1
COX57.2                  .......... .......... .......... .......... .......... .......G.. .......... ..A....... .......... ......C...   COX57.2
COX57.3                  .......... .......... .......... .......... .......... .......G.. .......... .......... .......... ......C...   COX57.3
COX57.4                  .......... .......... .......... .......... .......... .......G.. .......... .......... .......... ......C...   COX57.4
COX57.5                  .......... .......... .......... .......... .......... .......G.. .......... .......... .......... ......C...   COX57.5
COX57.6                  .......... .......... .......... .......... .......... .......G.. .......... .......... .......... ..........   COX57.6
COX57.7                  .......... .......... .......... .......... .......... .......G.. .......... .......... .......... ..........   COX57.7
COX57.8                  ---------- -......... .......... .......... .......... .......G.. .......... .......... .......... ..........   COX57.8
COX57.9                  .......... .......... .......... .......... .......... .......G.. .......... .......... .......... ......C...   COX57.9
COX57.10                 ---------- ---------- .......... .......... .......... .......G.. .......... .......... .......... ..........   COX57.10
COX57.Dugway_5J108-111   .......... .......... .......... .......... .......... .......G.. .......... .......... .......... ......C...   COX57.Dugway_5J108-111

                                110        120        130        140        150        160        170        180        190        200
                         ....|....| ....|....| ....|....| ....|....| ....|....| ....|....| ....|....| ....|....| ....|....| ....|....|
COX57.1                  GGCGGCCAGA TAGCTCAGTC GGTAGAGCAG AGGACTGAAA ATCCTCGTGT CGGCAGTTCG ATTCTGCCTC TGGCCACCAT GAAATCAAAC ACTTACCAAT   COX57.1
COX57.2                  .......... .......... .......... .......... .......... .......... .......... .......... .......... ..........   COX57.2
COX57.3                  .......... .......... .......... .......... .......... .......... .......... .......... .......... ..........   COX57.3
COX57.4                  .......... .......... .......... .......... .......... .......... .......... .......... .......... ..........   COX57.4
COX57.5                  .......... .......... .......... .......... .......... .......... .......... .......... .......... ..........   COX57.5
COX57.6                  .......... .......... .......... .......... .......... .......... .......... .......... .......... ..........   COX57.6
COX57.7                  .......... .......... .......... .......... .......... .......... .......... .......... .......... ..........   COX57.7
COX57.8                  .......... .......... .......... .......... .......... .......... .......... .......... .......... ..........   COX57.8
COX57.9                  .......... .......... .......... .......... .......... .......... .......... .......... .......... .A........   COX57.9
COX57.10                 .......... .......... .......... .......... .......... .......... .......... .......... .......... ..........   COX57.10
COX57.Dugway_5J108-111   .......... .......... .......... .......... .......... .......... .......... .......... .......... ..........   COX57.Dugway_5J108-111

                                210        220        230        240        250        260        270        280        290        300
                         ....|....| ....|....| ....|....| ....|....| ....|....| ....|....| ....|....| ....|....| ....|....| ....|....|
COX57.1                  TTTACCAGTT GGTTAAAATT TGCCCTTTTG GGCGTTTTGT GGAACGACAA CTGTGCATCA CGCTTCACCA AACTTAAACG TTGGACTACT ATTACTTGTC   COX57.1
COX57.2                  .......T.. .A.C...... ..A....G.. .......... .......... .......... .......... .......... .......... ..........   COX57.2
COX57.3                  .......... ...C...... ..G....G.. .......... .......... .......... .......... .......... .......... ..........   COX57.3
COX57.4                  .......T.. .A.C...... ..A....G.. .......... .......... .......... .......... .......... .......... ..........   COX57.4
COX57.5                  .......... .A.C...... ..A....G.. .......... .......... .......... .......... .......... .......... ..........   COX57.5
COX57.6                  .......... ...C...... ..A....G.. .......... .......... .......... .......... .......... .......... ..........   COX57.6
COX57.7                  .......... ...C...... ..A....G-. .......... .......... .......... .......... .......... .......... ..........   COX57.7
COX57.8                  .......... ...C...... ..A....G.. .......... .......... .......... .......... .......... .......... ..........   COX57.8
COX57.9                  .......... .A.C...... ..A....G.. .......... .......... .......... .......... .......... .......... ..........   COX57.9
COX57.10                 .......... ...C...... ..G....G.. .......... .......... .......... .......... .......... .......... ..........   COX57.10
COX57.Dugway_5J108-111   .......... ...C...... ..A....G.. .......... .......... .......... .......... .......... .......... ..........   COX57.Dugway_5J108-111

                                310        320        330        340        350        360        370        380        390        400
                         ....|....| ....|....| ....|....| ....|....| ....|....| ....|....| ....|....| ....|....| ....|....| ....|....|
COX57.1                  GGATAACAAG CTTTATTTGC CGACTTGATT AGGATTATCT CTATTTAAAT TACCGATTAA AATTAAAGAT ATCTGACTAA CTGATTAGCA AATTT-AACA   COX57.1
COX57.2                  .......... .......... ......A... .......... .......... .......... .......... .......... .......... .....T....   COX57.2
COX57.3                  .......... .......... .......... .......... .......... .......... .......... .......... .......... .....T....   COX57.3
COX57.4                  .......... .......... ......A... .......... .......... .......... .......... .......... .......... .....T....   COX57.4
COX57.5                  .......... .......... ......A... .......... .......... .......... .......... .......... .......... .....T....   COX57.5
COX57.6                  .......... .......... .......... .......... .......... .......... .......... .......... .......... .....T....   COX57.6
COX57.7                  .......... .......... .......... .......... .......... .......... .......... .......... .......... .....T....   COX57.7
COX57.8                  .......... .......... .......... .......... .......... .......... .......... .......... .......... .....T....   COX57.8
COX57.9                  .......... .......... ......A... .......... .......... .......... .......... .......... .......... .....T....   COX57.9
COX57.10                 .......... .......... .......... .......... .......... .......... .......... .......... .......... .....T....   COX57.10
COX57.Dugway_5J108-111   .......... .......... ......A... .......... .......... .......... .......... .......... .......... .....T....   COX57.Dugway_5J108-111

                                410        420        430        440        450        460        470        480        490        500
                         ....|....| ....|....| ....|....| ....|....| ....|....| ....|....| ....|....| ....|....| ....|....| ....|....|
COX57.1                  TTTCAAAATA ATACTTATAA TCTATTTCCA AATCGCTATT ACTTGATATT TAAGTCTAAA ACAGATGATA AAAAATTAAT TTAAAAATCA CTAAAAAGTG   COX57.1
COX57.2                  .......... .......... .......... .......... .......... .......... .......... .......... .......... ..........   COX57.2
COX57.3                  .......... .......... .......... .......... .......... .......... .......... .......... .......... ..........   COX57.3
COX57.4                  .......... .......... .......... .......... .......... .......... .......... .......... .......... ..........   COX57.4
COX57.5                  .......... .......... .......... .......... .......... .......... .......... .......... .......... ..........   COX57.5
COX57.6                  .......... .......... .......... .......... .......... .......... .......... .......... .......... ..........   COX57.6
COX57.7                  .......... .......... .......... .......... .......... .......... .......... .......... .......... ..........   COX57.7
COX57.8                  .......... .......... .......... .......... .......... .......... .......... .......... .......... ..........   COX57.8
COX57.9                  .......... .......... .......... .......... .......... .......... .......... .......... .......... ..........   COX57.9
COX57.10                 .......... .......... .......... .......... .......... .......... .......... .......... .......... ..........   COX57.10
COX57.Dugway_5J108-111   .......... .......... .......... .......... .......... .......... .......... .......... .......... ..........   COX57.Dugway_5J108-111

                                510        520        530        540        550        560        570        580        590        600
                         ....|....| ....|....| ....|....| ....|....| ....|....| ....|....| ....|....| ....|....| ....|....| ....|....|
COX57.1                  ACAAATTATA AAAATTGATT TTAAAAATAA TTTTTACAAA CTAGCACTAA CGAGTATAAT TTTGATAAAT AATAATAAAT ACCGCCAAAG GCTTACGCCT   COX57.1
COX57.2                  .......... .......... .......... .......... .......... .......... .......... .......... .......... ..........   COX57.2
COX57.3                  .......... .......... .......... .......... .......... .......... .......... .......... .......... ..........   COX57.3
COX57.4                  .......... .......... .......... .......... .......... .......... .......... .......... .......... ..........   COX57.4
COX57.5                  .......... .......... .......... .......... .......... .......... .......... .......... .......... ..........   COX57.5
COX57.6                  .......... .......... .......... .......... .......... .......... .......... .......... .......... ..........   COX57.6
COX57.7                  .......... .......... .......... .......... .......... .......... .......... .......... .......... ..........   COX57.7
COX57.8                  .......... .......... .......... .......... .......... .......... .......... .......... .......... .....-----   COX57.8
COX57.9                  .......... .......... .......... .......... .......... .......... .......... .......... .......... ..........   COX57.9
COX57.10                 .......... .......... .......... .......... .......... .......... .......... .......... ......---- ----------   COX57.10
COX57.Dugway_5J108-111   .......... .......... .......... .......... .......... .......... .......... .......... .......... ..........   COX57.Dugway_5J108-111

                         ....|.
COX57.1                  TCCACC   COX57.1
COX57.2                  ......   COX57.2
COX57.3                  ......   COX57.3
COX57.4                  ......   COX57.4
COX57.5                  ......   COX57.5
COX57.6                  ......   COX57.6
COX57.7                  ......   COX57.7
COX57.8                  ------   COX57.8
COX57.9                  ......   COX57.9
COX57.10                 ------   COX57.10
COX57.Dugway_5J108-111   ......   COX57.Dugway_5J108-111
```

```
                                10         20         30         40         50         60         70         80         90        100
COX61.1                 CAGTAGCAAA TTCGGAATAC CCCATTTTTC CTTTTACTTT CACGTATCGA TAAGCATCGC TTTCCAAGAA TTTATTGGCA TAGGAGACCT TCCCATCCTG COX61.1
COX61.2                 .......... .......... ........G. .....G.... .......... .......... .......... .......... .......... .T.......A COX61.2
COX61.3                 .......... .......... ........G. .....G.... .......... .......... .......... .......... .......... .........A COX61.3
COX61.4                 .......... .......... ........G. .....G.... .......... .......... .......... .......... .......... .......... COX61.4
COX61.5                 .......... .......... ........G. .....G.... .......... .......... .......... .......... .......... .........A COX61.5
COX61.6                 .......... .......... ........G. .....G.... .......... .......... .......... .......... .......... .......... COX61.6
COX61.7                 .......... .......... ........G. .....G.... .......... .......... .......... .......... .......... .........A COX61.7
COX61.8                 .......... .......... ...G....G. .....G.... .......... .......... .......... .......... .......... .........A COX61.8
COX61.9                 .......... .......... ........G. .....G.... .......... .......... .......... .......... .......... .T.......T COX61.9
COX61.Dugway_5J108-111  .......... .......... ........G. .....G.... .......... .......... .......... .......... .......... .......... COX61.Dugway_5J108-111

                               110        120        130        140        150        160        170        180        190        200
COX61.1                 AAACGCAAAC TGATGCA-CA TAGCAAATCC ATCAAACCAG TGCCGCAGTT TTTTATTACC TGCTTCAAAC TTAGCCGGAC CGTTTCGAAT CAGCGTCCCA COX61.1
COX61.2                 .......... .......A.. .......... .......... .......... .......... .......... .....T.... .......... .......... COX61.2
COX61.3                 .......... .......A.. .......... .......... .......... .......... .......... .......... .......... .......... COX61.3
COX61.4                 .......... .......A.. .......... .......... .......... .......... .......... .......... .......... .......... COX61.4
COX61.5                 .......... .......A.. .......... .......... .......... .......... .......... .......... .......... .......... COX61.5
COX61.6                 .......... .......A.. .......... .......... .......... .......... .......... .......... .......... .......... COX61.6
COX61.7                 .......... .......A.. .......... .......... .......... .......... .......... .......... .......... .......... COX61.7
COX61.8                 .......... .......A.. .......... .......... .......... .......... .......... .......... .......... .......... COX61.8
COX61.9                 .......... .......A.. .......... .......... .......... .......... .......... .....T.... .......... .......... COX61.9
COX61.Dugway_5J108-111  .......... .......A.. .......... .......... .......... .......... .......... .......... .......... .......... COX61.Dugway_5J108-111

                               210        220        230        240        250        260        270        280        290        300
COX61.1                 GAAAGCCATT CTGGCAATGC GCCTTCAATC GGTAACTCCG AAATGACCAC TTCGTTGTTC AAGGTTGAAA AACCTAAGGA ATACTTTCTA TTCATAAAGA COX61.1
COX61.2                 .....T.... .......... .....T.... .......... .......... .......... .......... .......... .......... .......... COX61.2
COX61.3                 .....T.... .......... .....T.... .......... .......... .......... .......... .......... .......... .......... COX61.3
COX61.4                 .......... .......... .......... .......... .......... .......... .......... .......... .......... .......... COX61.4
COX61.5                 .....T.... .......... .....T.... .......... .......... .......... .......... .......... .......... .......... COX61.5
COX61.6                 .......... .......... .......... .......... .......... .......... .......... .......... .......... .......... COX61.6
COX61.7                 .....T.... .......... .....T.... .......... .......... .......... .......... .......... .......... .......... COX61.7
COX61.8                 .....T.... .......... .....T.... .......... .......... .......... .......... .......... .......... .......... COX61.8
COX61.9                 .....T.... .......... .....T.... .......... .......... .......... .......... .......... .......... .......... COX61.9
COX61.Dugway_5J108-111  .......... .......... .......... .......... .......... .......... .......... .......... .......... .......... COX61.Dugway_5J108-111

                               310        320        330        340        350        360        370        380        390        400
COX61.1                 TAAGATTACC ACTTACCTCC GATTGAGTCG AACCCTTAGT TTTTCGTTAG TTCAAAAGAT TGACATTATC GAACAAACCT AGCTATTTTA AAAACCGAAA COX61.1
COX61.2                 .......... .......... .......... .......... .......... .......... .......... .......... .......... .......... COX61.2
COX61.3                 .......... .......... .......... .......... .......... .......... .......G.. .......... .......... .......... COX61.3
COX61.4                 .......... .......... .......... .......... .......... .......... .......... .......... .......... .......... COX61.4
COX61.5                 .......... .......... .......... .......... .......... .......... .......... .......... .......... .......... COX61.5
COX61.6                 .......... .......... .......... .......... .......... .......... .......... .......... .......... .......... COX61.6
COX61.7                 .......... .......... .......... .......... .......... .......... .......... .......... .......... .......... COX61.7
COX61.8                 .......... .......... .......... .......... .......... .......... .......G.. .......... .......... .......... COX61.8
COX61.9                 .......... .......... .......... .......... .......... .......... .......... .......... .......... .......... COX61.9
COX61.Dugway_5J108-111  .......... .......... .......... .......... .......... .......... .......... .......... .......... .......... COX61.Dugway_5J108-111

                               410        420        430        440        450        460        470        480        490        500
COX61.1                 AGGCAAAAAC TGATCGCCAC CCCACTTACT TTACGTAATT TAAAAAATAA GCTAAGTTTA GGCCCCCTCT CATAAAAACC GGAGGTTCAA ATGCCATTAT COX61.1
COX61.2                 .......... .......... .......... .......... .......... .......... .......... .......... .......... .......... COX61.2
COX61.3                 .......... .......... .......... .......... .......... .......... .......... .......... .......... .......... COX61.3
COX61.4                 .......... .......... ......C... .......... .......... A.......C. .......... .......... .......... .......... COX61.4
COX61.5                 .......... .......... .......... .......... .......... .......... .......... .......... .......... .......... COX61.5
COX61.6                 .......... .......... ......C... .......... .......... ........C. .......... .......... .......... .......... COX61.6
COX61.7                 .......... .......... .......... .......... .......... .......... .......... .......... .......... .......... COX61.7
COX61.8                 .......... .......... .......... .......... .......... .......... .......... .......... .......... .......... COX61.8
COX61.9                 .......... .......... .......... .......... .......... .......... .......... .......... .......... .......... COX61.9
COX61.Dugway_5J108-111  .......... .......... ......C... .......... .......... .......... .......... .......... .......... .......... COX61.Dugway_5J108-111

                               510        520        530        540        550        560        570
COX61.1                 TAACCTACAA GTTTGATAGA AATTTAAATA GCGAGAAAGT TTTTCAATAC ACTCCGCCCC CAGAAATCAA COX61.1
COX61.2                 .......... .......... .......... .......... .......... .......... .......... COX61.2
COX61.3                 .......... .......... .......... .......... .......... .......... .......... COX61.3
COX61.4                 .......... .......... .......... .......... .......... .......... .......... COX61.4
COX61.5                 .......... .......... .......... .......... .......... .......... .......... COX61.5
COX61.6                 .......... .......... .......... .......... .......... .......... .......... COX61.6
COX61.7                 .......... .......... .......... .......... .......... ......T... .......... COX61.7
COX61.8                 .......... .......... .......... .......... .......... .......... .......... COX61.8
COX61.9                 .......... .......... .......... .......... .......... .......... .......... COX61.9
COX61.Dugway_5J108-111  .......... .......... .......... .......... .......... .......... .......... COX61.Dugway_5J108-111
```
