# Supplementary figures and images for: Rapid Typing of Coxiella burnetii
Source: PLoS One. 2011 Nov 2;6(11):e26201. doi: 10.1371/journal.pone.0026201 (PMC3206805; doi:10.1371/journal.pone.0026201)

A: full tree

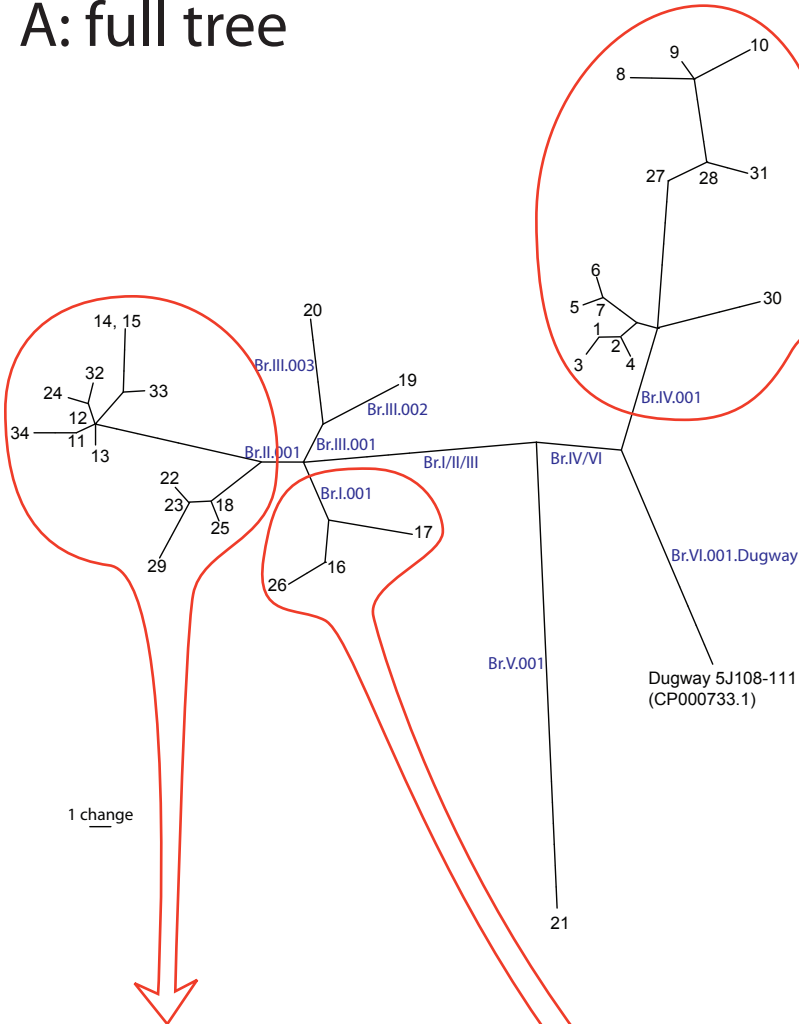

B: genomic group IV

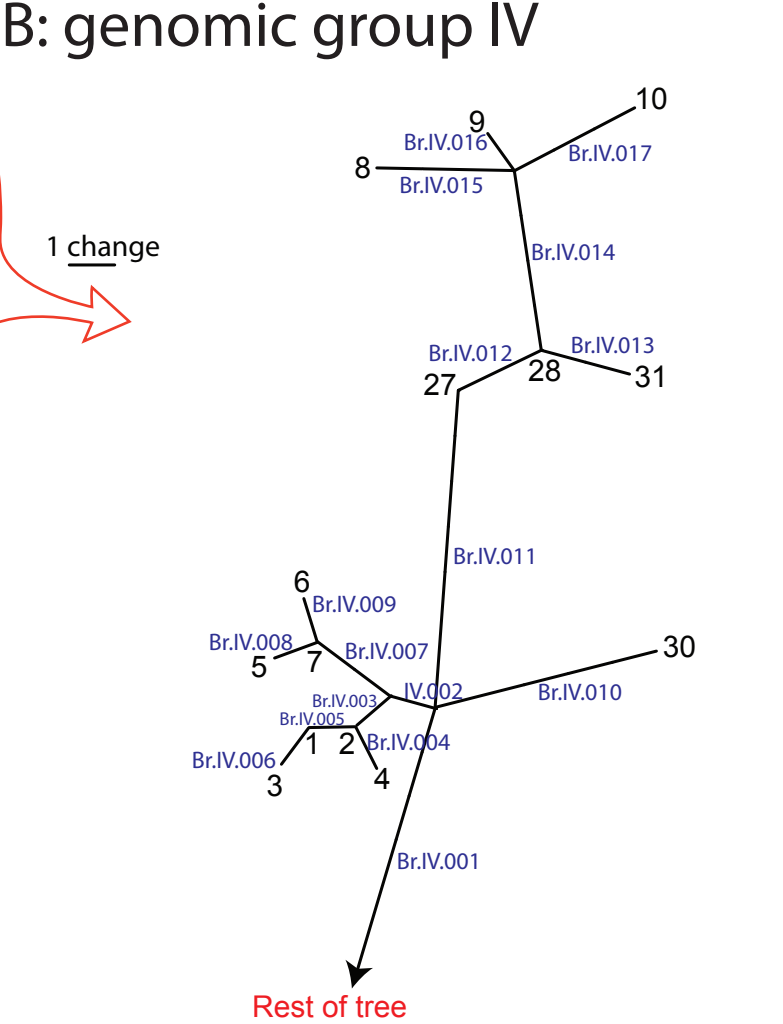

C: genomic group II

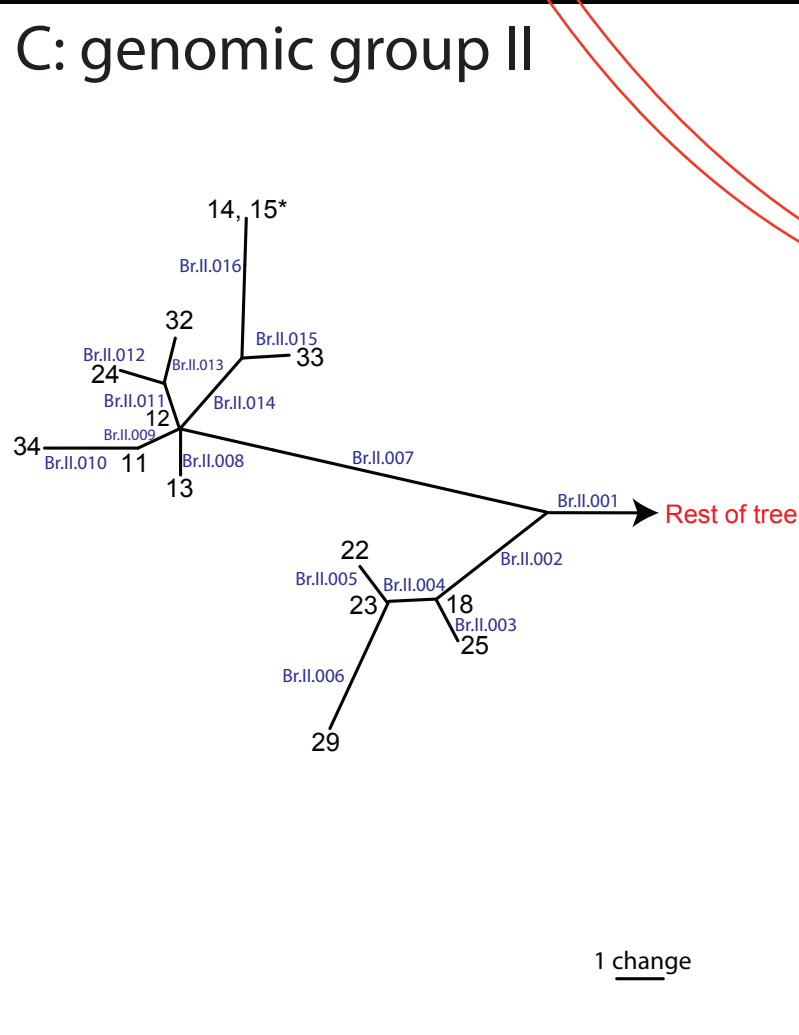

D: genomic group I

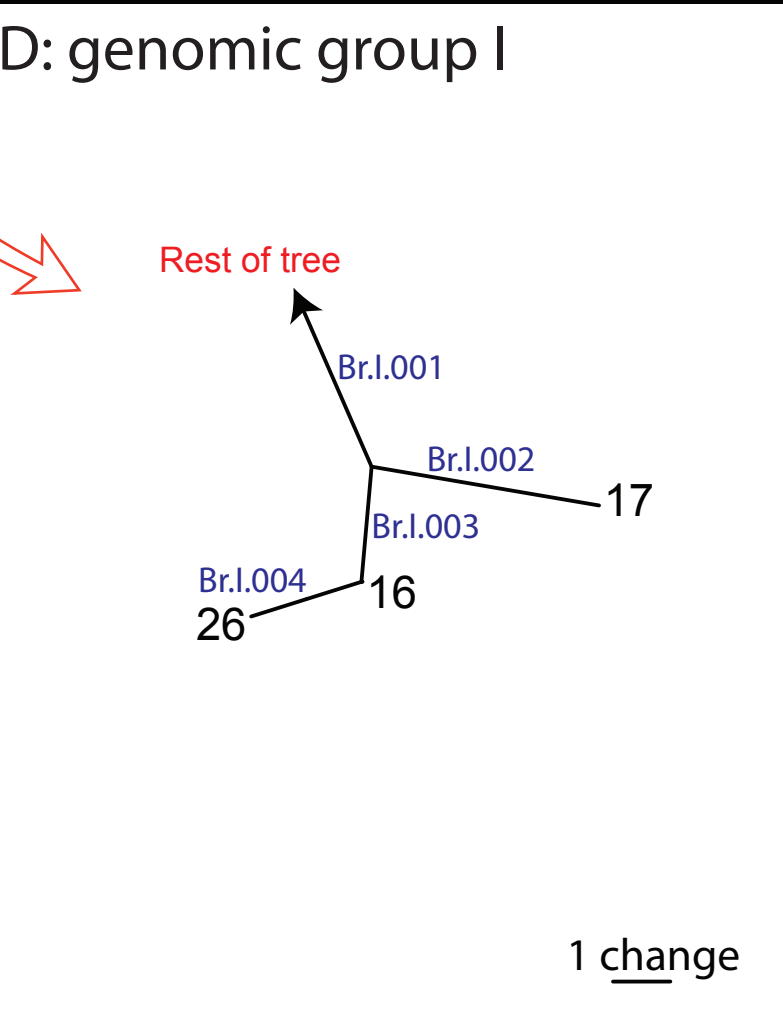

Supplement: Figure S2 — Phylogenetic tree of MST genotypes with labeled branches. Panel A: complete tree as in Figure 1. Panels B–C provide an expanded view of different groups to better visualize branch names; branch names were assigned based on the genomic group nomenclature (I–VI) described in Hendrix et al. [10] and can be used with Table S1 to determine the location of each of the 112 loci on this tree. (PDF) [file pone.0026201.s002.pdf]
